# Complete plastomes of six species of *Wikstroemia* (Thymelaeaceae) reveal paraphyly with the monotypic genus *Stellera*

**DOI:** 10.1038/s41598-021-93057-3

**Published:** 2021-06-30

**Authors:** Liefen He, Yonghong Zhang, Shiou Yih Lee

**Affiliations:** 1 School of Life Sciences, Yunnan Normal University, Kunming, China; 2 State Key Laboratory of Biocontrol and Guangdong Provincial Key Laboratory of Plant Resources, School of Life Sciences, Sun Yat-sen University, Guangzhou, China

**Keywords:** Plant sciences, Systems biology

## Abstract

*Wikstroemia* (Thymelaeaceae) is a diverse genus that extends from Asia to Australia and has been recorded on the Hawaiian Islands. Despite its medicinal properties and resource utilization in pulp production, genetic studies of the species in this important genus have been neglected. In this study, the plastome sequences of six species of *Wikstroemia* were sequenced and analysed. The plastomes ranged in size between 172,610 bp (*W. micrantha*) and 173,697 bp (*W. alternifolia*) and exhibited a typical genome structure consisting of a pair of inverted repeat (IR) regions separated by a large single-copy (LSC) region and a small single-copy (SSC) region. The six plastomes were similar in the 138 or 139 genes predicted, which consisted of 92 or 93 protein-coding genes, 38 tRNA genes, and 8 rRNA genes. The overall GC contents were identical (36.7%). Comparative genomic analyses were conducted with the inclusion of two additional published species of *Wikstroemia* in which the sequence divergence and expansion of IRs in the plastomes were determined. When compared to the coding sequences (CDSs) of *Aquilaria sinensis*, five genes, namely, *rpl*2, *rps*7, *rps*18, *ycf*1 and *ycf*2, indicated positive selection in *W. capitata*. The plastome-based phylogenetic analysis inferred that *Wikstroemia* in its current state is paraphyletic to *Stellera chamaejasme*, while the ITS-based tree analyses could not properly resolve the phylogenetic relationship between *Stellera* and *Wikstroemia*. This finding rekindled interest in the proposal to synonymize *Stellera* with *Wikstroemia*, which was previously proposed but rejected due to taxonomic conflicts. Nevertheless, this study provides valuable genomic information to aid in the taxonomic implications and phylogenomic reconstruction of Thymelaeaceae.

## Introduction

*Wikstroemia* Engl. (Thymelaeaceae) is a diverse genus of approximately 70 species. Members of *Wikstroemia* are widely distributed in the Asian and Oceanian regions and scattered around the Hawaiian Islands^1^. The species are mostly fibrous trees, shrubs or subshrubs with a woody rhizome. Several species are cultivated as raw material for pulp production^2,3^, while a handful of them are reported to have medicinal properties^4,5^. However, studies of *Wikstroemia* have been confined to its utilization in pulp production and pharmacological applications; reports on genetic studies of *Wikstroemia* are scarce.

The only reports on the genetic diversity to date include one on *Wikstroemia ganpi* in Korea using inter simple sequence repeat (ISSR) markers^6^ and two, published, complete plastome sequences of *Wikstroemia chamaedaphne* and *Wikstroemia indica*^7,8^. Due to the lack of molecular evidence, taxonomic studies of *Wikstroemia* have relied solely on morphological characteristics^9^. Ironically, the continuous nature of morphological variation in members of *Wikstroemia* has led to much taxonomic confusion in attempts to distinguish species and has resulted in ambiguities in taxonomic classifications between *Wikstroemia* and its sister genera^9,10^. Among the key morphological characteristics proposed to differentiate *Wikstroemia* from allied genera is the presence of petaloid scales in the flower^11^. However, the presence and characteristics of the disc in the flowers of *Wikstroemia* was not emphasized. Failure to analyse this character may result in misidentifications due to overlap in this feature during classification^10^. The subgeneric classification of *Wikstroemia*, consisting of only the subgenera *Wikstroemia* and *Diplomorpha*, is generally accepted^12,13^. Another problem in classification is the difficulty in detecting natural hybridization among the species due to the possibility of low reproductive isolation and high genetic similarity, suggesting that *Wikstroemia* represents a large complex of species^14^.

The plastome is a circular double-stranded DNA molecule. In plants, the plastome is mostly maternally inherited and not disturbed by gene recombination^15^. A typical plant plastome ranges in size from 120 to 217 kb^16^. The complete plastome has a typical quadripartite structure, including a large single-copy (LSC) region, a small single-copy (SSC) region, and two separate inverted regions (IRs)^17^. Owing to its slow rate of evolution and ease of sequencing and assembly due to its small size, the plastome has been receiving much attention among biologist and taxonomist because it is highly informative and provides evolutionary and genetics insights^18,19^.

The taxonomic placement of *Wikstroemia* has been controversial. This genus has experienced a complicated classification history in reviews of members of the Thymelaeaceae. *Stellera chamaejasme* of the monotypic genus *Stellera* was reported to be sister to *Wikstroemia* based on combined plastid DNA sequences (*trn*T-*trn*L, *trn*L-*trn*F, *trn*L intron, and *rpl*16 intron)^20^, while *Wikstroemia*, along with 14 sister genera based on palynology findings, has been taxonomically placed in the Daphne group of the tribe Daphneae^21,22^. Although phylogenetic studies in Thymelaeaceae are ongoing^23^, phylogenetic relationships in *Wikstroemia* are likely to be understudied. Constituent genera in Thymelaeaceae have experienced similar molecular challenges, in which poor phylogenetic resolution is likely due to low genetic variation in the selected molecular markers^23^. Such conflicts can be overcome by utilizing genome-scale datasets^24^. At the same time, highly divergent regions may be identified through genome comparisons, which could aid in future phylogenetic studies of such a diverse genus as *Wikstroemia*.

In this study, we sequenced the complete plastomes of six species of *Wikstroemia*, *W. alternifolia, W. canescens, W. capitata, W. dolicantha, W. micrantha*, and *W. scytophylla*, to analyse and compare genomes using bioinformatic tools. Our aims were to (1) characterize the plastomes of the six species of *Wikstroemia*; (2) examine the variation in sequence repeats and codon usage in the six plastome sequences; (3) identify highly divergent regions in the plastome sequences; and (4) improve the understanding of the intrageneric/intergeneric phylogeny of *Wikstroemia* within Thymelaeaceae based on plastome sequences and the nuclear ribosomal DNA internal transcribed spacer (ITS) region.

## Results

### Plastome features of six species of *Wikstroemia*

**Figure 1:**
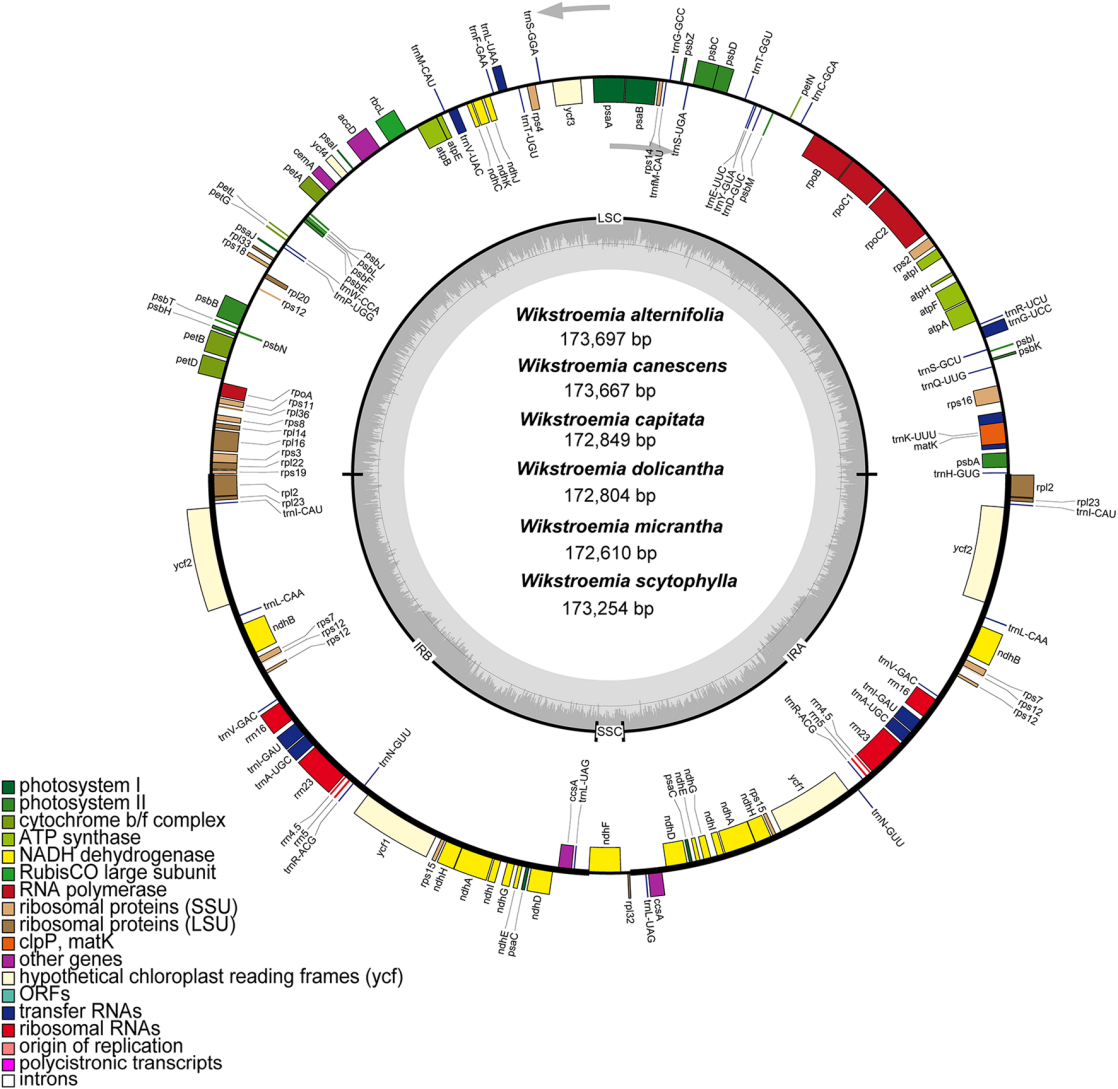
Gene map for the plastomes of six species of *Wikstroemia* used in this study. Genes on the inside of the map are transcribed in the clockwise direction; genes on the outside of the map are transcribed in the counterclockwise direction. Darker grey in the inner circle represents the GC content, whereas light grey corresponds to the AT content. Different functional groups of genes are shown in different colours. The gene map was generated using OGDRAW^45^.

The total length of the plastomes of the six species of *Wikstroemia* analysed in this study ranged from 172,610 bp (*W. micrantha*) to 173,697 bp (*W. alternifolia*). All six plastomes exhibited a typical quadripartite structure (Table 1, Fig. 1) consisting of a pair of inverted repeat (IR) regions (41,850–42,073 bp) separated by an LSC region (86,111–86,701 bp) and an SSC region (2799–2871 bp). All six plastomes had the same GC content at 36.7%. However, the GC content in the plastome of each species of *Wikstroemia* was unevenly distributed. The IR region accounted for the highest GC content (38.8–38.9%), followed by the LSC region (34.8–34.9%), while the SSC region showed the lowest GC content (28.7–29.6%).

**Table 1:** Plastome features of six species of *Wikstroemia.*

Species	Origin	Collector and collection number	Coordinates (longitude, latitude)	Plastome								Plastid genes				GenBank accession number	
				Total (bp)	GC content (%)	LSC (bp)	GC content (%)	SSC (bp)	GC content (%)	IR (bp)	GC content (%)	Total	CDS	tRNA	rRNA	Plastome	ITS
*Wikstroemia alternifolia*	Batang County, Sichuan	Y. H. Zhang et al., RXK30	29°19′27″N, 99°18′40″E	173,697	36.7	86,694	34.8	2857	29.5	42,073	38.8	139	93	38	8	MW073913	MW075476
*Wikstroemia canescens*	Batang County, Sichuan	Y. H. Zhang et al., RXK32	29°19′27″N, 99°18′40″E	173,667	36.7	86,701	34.8	2854	29.6	42,056	38.8	139	93	38	8	MW073911	MW075477
*Wikstroemia capitata*	Guanyang, Wushan County, Chongqing	Y. H. Zhang and W. G. Sun, RXK33	31°28′02″N, 109°55′53″E	172,849	36.7	86,154	34.8	2871	29.4	41,912	38.9	138	92	38	8	MW073909	MW075480
*Wikstroemia dolicantha*	Kunming, Yunnan	Y. H. Zhang et al. RXK39	25°07′48″N, 102°42′24″E	172,804	36.7	86,230	34.8	2854	28.7	41,860	38.9	138	92	38	8	MW073912	MW075475
*Wikstroemia micrantha*	Changshou County, Chongqing	Y. H. Zhang et al. S0693-Zhang4	29°49′02″N, 107°4′35″E	172,610	36.7	86,111	34.9	2799	29.5	41,850	38.9	139	93	38	8	MN756675	MW075479
*Wikstroemia scytophylla*	Kunming Botanical Garden	Y. H. Zhang, et al. RXK48	25°08′36″N, 102°44′27″E	173,254	36.7	86,338	34.8	2840	29.4	42,038	38.8	139	93	38	8	MW073910	MW075474

The six plastomes of *Wikstroemia* displayed an identical gene content and gene order with no structural reconfigurations. A total of 138 to 139 genes were detected in the six species used in this study, comprising 92 to 93 protein-coding genes , 38 transfer RNA (tRNA) genes, and 8 ribosomal RNA (rRNA) genes (Table 1). However, 27 genes were duplicated in the IR regions, including 15 protein-coding genes (*ccs*A, *ndh*A, *ndh*B, *ndh*D, *ndh*E, *ndh*H, *ndh*G, *ndh*I, *psa*C, *rpl*2, *rpl*23, *rps*7, *rps*15, *ycf*1, *ycf*2), eight tRNA genes (*trn*A-UGC, *trn*I-CAU, *trn*I-GAU, *trn*L-CAA, *trn*L-UAG, *trn*N-GUU, *trn*R-ACG and *trn*V-GAC) and four rRNAs (*rrn*4.5, *rrn*5, *rrn*16 and *rrn*23) (Table 2). Fifteen genes contained an intron, five of which (*ndh*A, *ndh*B, *rpl*2, *trn*A-UGC and *trn*I-GAU) were located in the IR region, and the remaining 10 genes (*atp*F, *pet*B, *pet*D, *rpl*16, *rpo*C1, *rps*16, *trn*G-UCC, *trn*L-UAA, *trn*K-UUU and *trn*V-UAC) were located in the LSC region (see Supplementary Table S1 online). Only the *ycf*3 gene, which was present in the LSC region, was detected to contain a pair of introns. Upon comparison, we found that the *trn*K-UUU gene had the longest intron, ranging from 2498 to 2508 bp, in all six genomes.

**Table 2:** Genes present in the plastomes of six species of *Wikstroemia* used in this study.

	Genes
RNAs, ribosomal	*rrn*4.5(×2), *rrn*5(×2), *rrn*16(×2)*, rrn*23(×2)
RNAs, transfer	*trn*A-UGC(×2), *trn*C-GCA, *trn*D-GUC, *trn*E-UUC, *trn*F-GAA, *trn*fM-CAU, *trn*G-GCC, *trn*G-UCC, *trn*H-GUG, *trn*I-CAU(×2), *trn*I-GAU(×2), *trn*K-UUU, *trn*L-CAA(×2), *trn*L-UAA, *trn*L-UAG(×2), *trn*M-CAU, *trn*N-GUU(×2), *trn*P-UGG, *trn*Q-UUG, *trn*R-ACG(×2), *trn*R-UCU, *trn*S-GCU, *trn*S-GGA, *trn*S-UGA, *trn*T-GGU, *trn*T-UGU, *trn*V-GAC(×2), *trn*V-UAC, *trn*W-CCA, *trn*Y-GUA
Transcription and splicing	*mat*K, *rpo*A, *rpo*B, *rpo*C1, *rpo*C2
Small subunit	*rps*3, *rps*4, *rps*7(×2), *rps*8, *rps*11, *rps*12, *rps*14, *rps*15(×2), *rps*16, *rps*18, *rps*19
Large subunit	*rpl*2(×2), *rpl*14, *rpl*16, *rpl*20, *rpl*22, *rpl*23(×2), *rpl*32, *rpl*33, *rpl*36
ATP synthase	*atp*A, *atp*B, *atp*E, *atp*F, *atp*H, *atp*I
Photosystem I	*psa*A, *psa*B, *psa*C(×2), *psa*I, *psa*J
Photosystem II	*psb*A, *psb*B, *psb*C, *psb*D, *psb*E, *psb*F, *psb*H, *psb*I, *psb*J, *psb*K, *psb*L, *psb*M, *psb*N, *psb*T, *psb*Z
Calvin cycle	*rbc*L
Cytochrome complex	*pet*A, *pet*B, *pet*D*, pet*G, *pet*L, *pet*N
NADH dehydrogenase	*ndh*A(×2), *ndh*B(×2), *ndh*C, *ndh*D(×2), *ndh*E(×2), *ndh*F, *ndh*G(×2), *ndh*H(×2), *ndh*I(×2), *ndh*J, *ndh*K
Others	*acc*D, *ccs*A(×2), *cem*A, *ycf*1(×2), *ycf*2(×2), *ycf*3, *ycf*4

### Repetitive sequence analysis

**Figure 2:**
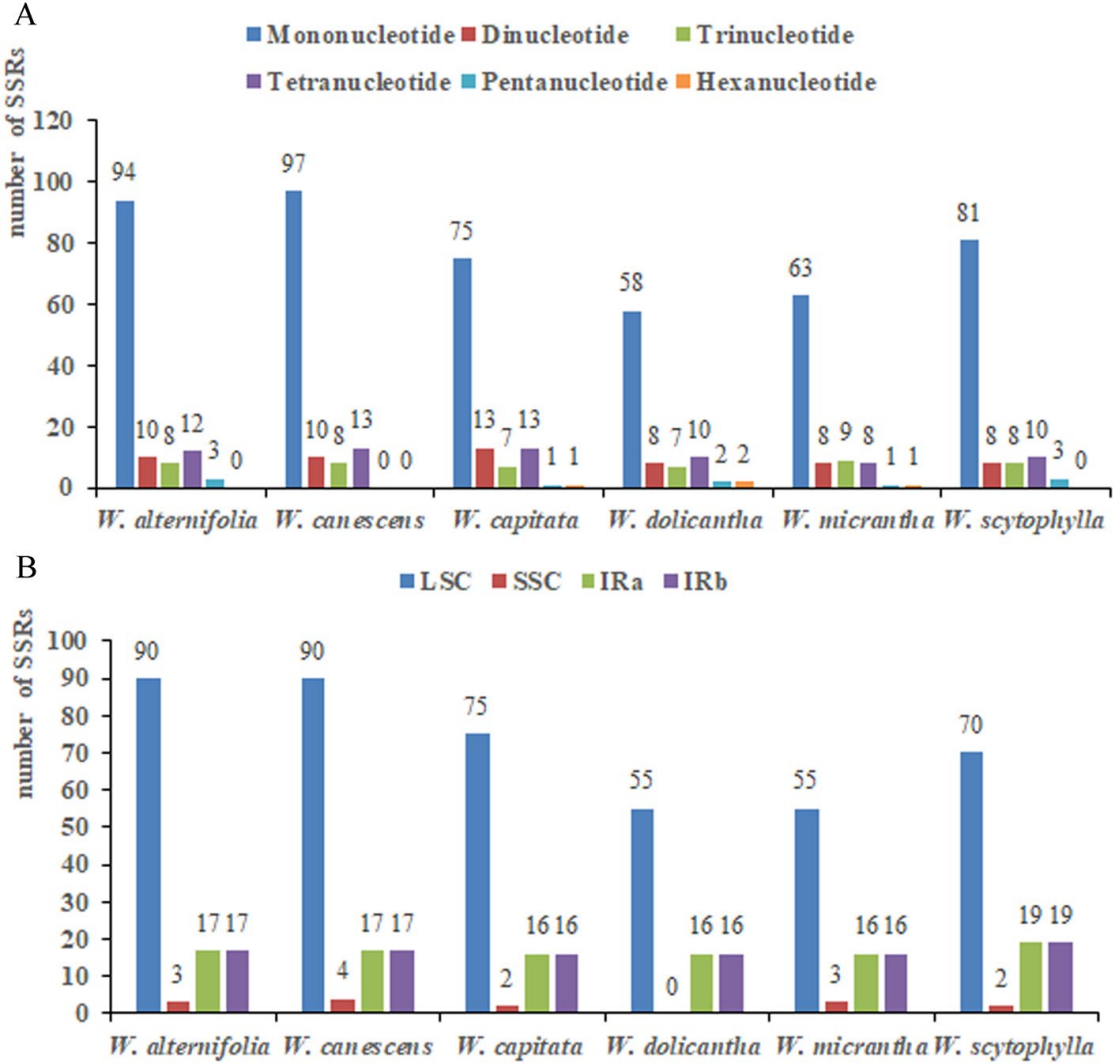
Distribution of small sequence repeats (SSRs) in the plastomes of six accessions of *Wikstroemia*. (**A**) Number of different SSR types detected in the plastomes of six species of *Wikstroemia*; (**B**) Frequencies of identified SSRs in large single-copy (LSC), small single-copy (SSC) and inverted repeat (IR) regions.

The total number of short sequence repeats (SSRs) in the plastome sequences of *W. alternifolia*, *W. canescens*, *W. capitata*, *W. dolicantha*, *W. micrantha*, and *W. scytophylla* were 127, 128, 110, 87, 90 and 110, respectively (Fig. 2A). No hexanucleotide sequences, however, were detected in the plastome sequences of *W. alternifolia*, *W. canescens* and *W. scytophylla*. The majority of SSRs (*W. alternifolia*: 70.87%; *W. canescens*: 70.31%; *W. capitata*: 68.18%; *W. dolicantha*: 63.22%; *W. micrantha*: 61.11%, *W. scytophylla*: 63.64%) were located in the LSC regions rather than in the other two regions of the plastome (Fig. 2B).

**Figure 3:**
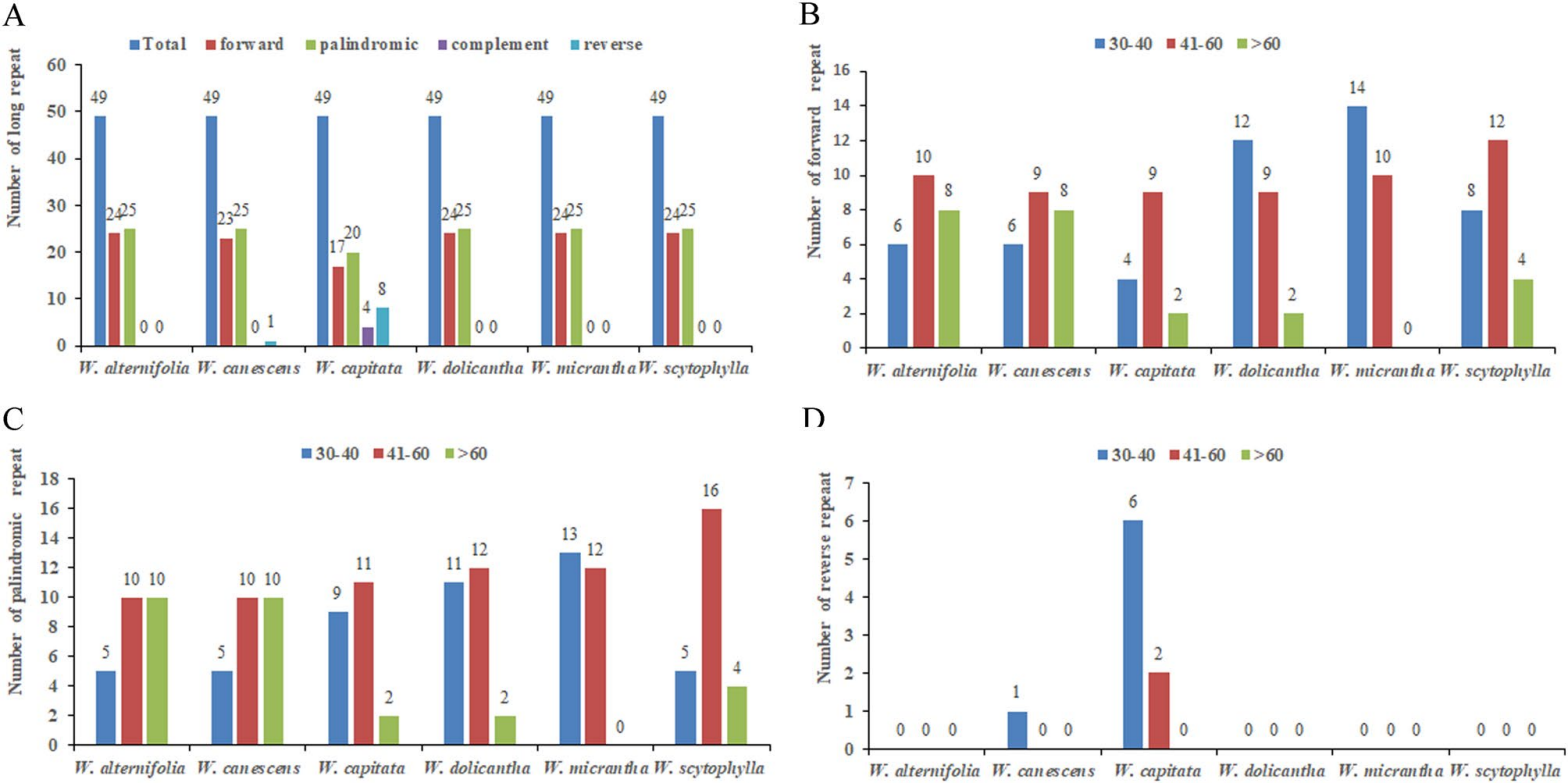
Analysis of long repeat sequences in the plastomes of six species of *Wikstroemia*. (**A**) Quantities of long repeats based on type; (**B**) frequencies of forward repeats by length; (**C**) frequencies of palindromic repeats by length; and (**D**) frequencies of reverse repeats by length.

All six species of *Wikstroemia* contained the same number of long repeats (Fig. 3A). In general, all of them contained 24 forward repeats and 25 palindromic repeats, except for *W. canescens* and *W. capitata*. Long forward repeats that ranged between 30 and 40 bp were the most abundant in *W. dolicantha* and *W. micrantha*, while *W. alternifolia*, *W. canescens*, *W. capitata*, and *W. scytophylla* were noted to have a higher number of long forward repeats with lengths of 41 to 60 bp (Fig. 3B). Long palindromic repeats were equally abundant in *W. alternifolia* and *W. canescens*, ranging from 40 to 60 bp and above 60 bp (Fig. 3C), while long palindromic repeats were abundant in the range of 30 to 60 bp in *W. capitata*, *W. dolicantha*, *W. micrantha* and *W. scytophylla*. Long reverse repeats, mostly within the range of 30 to 40 bp, were detected only in *W. canescens* and *W. capitata* (Fig. 3D).

### Analysis of codon usage

Thirty preferred codons (relative synonymous codon usage; RSCU > 1.00) were recorded in *W. alternifolia*, *W. canescens*, *W. capitata*, *W. dolicantha*, *W. micrantha* and *W. scytophylla* (see Supplementary Table S2 online). The stop codon UAA was most abundant and preferred over the other two stop codons, UAG and UGA, in all six species. Preferred codons mostly ended with the amino acids A or U, except for the leucine-encoded (Leu) codon UUG. The Leu-encoded codons had the greatest occurrence (9.38%), while cysteine-encoded (Cys) codons had the fewest occurrences (3.13%) among all six species of *Wikstroemia*.

### Sequence divergence analysis

**Figure 4:**
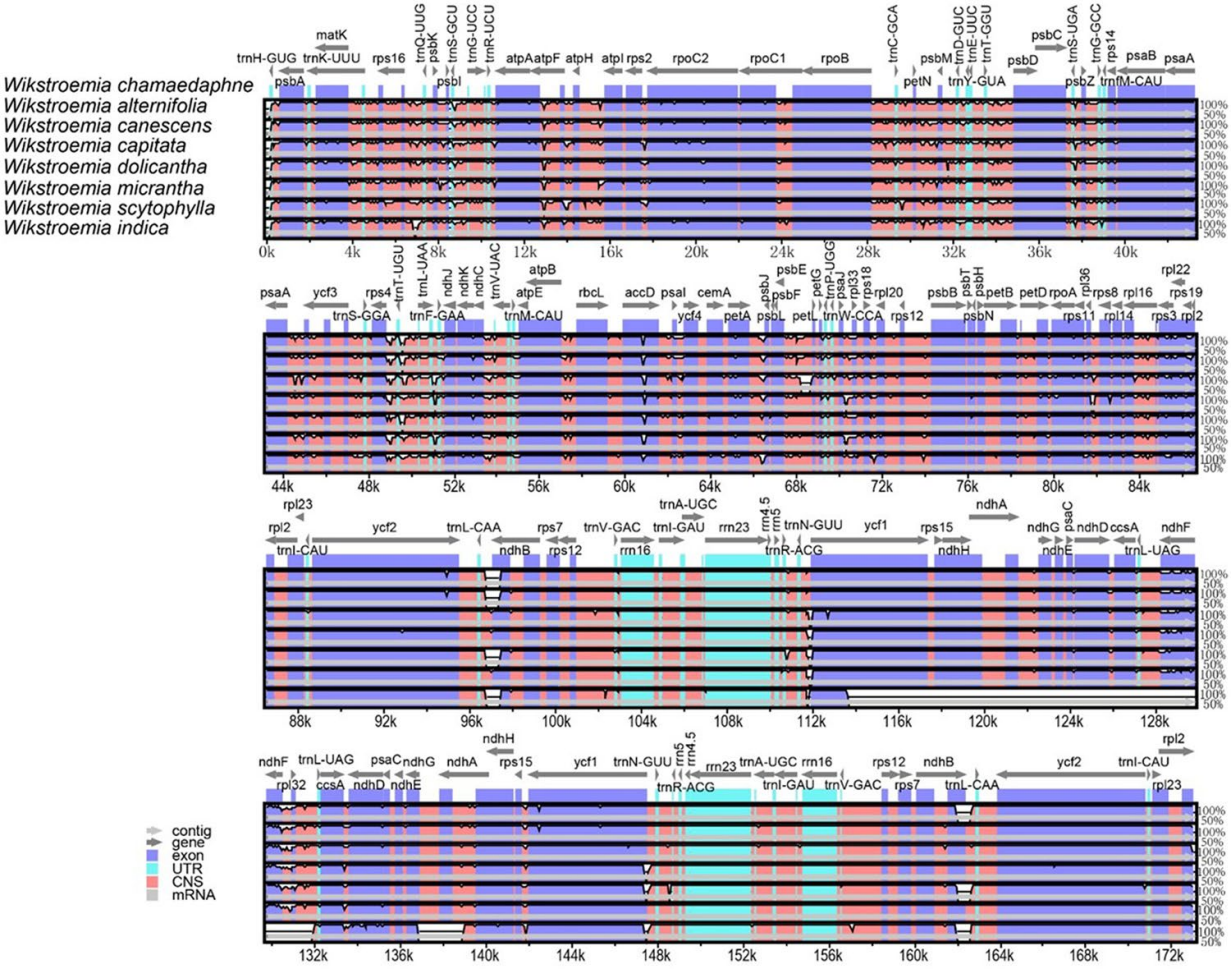
Complete plastome comparison of eight species of *Wikstroemia* using the plastome of *W. chamaedaphne* as reference.

**Figure 5:**
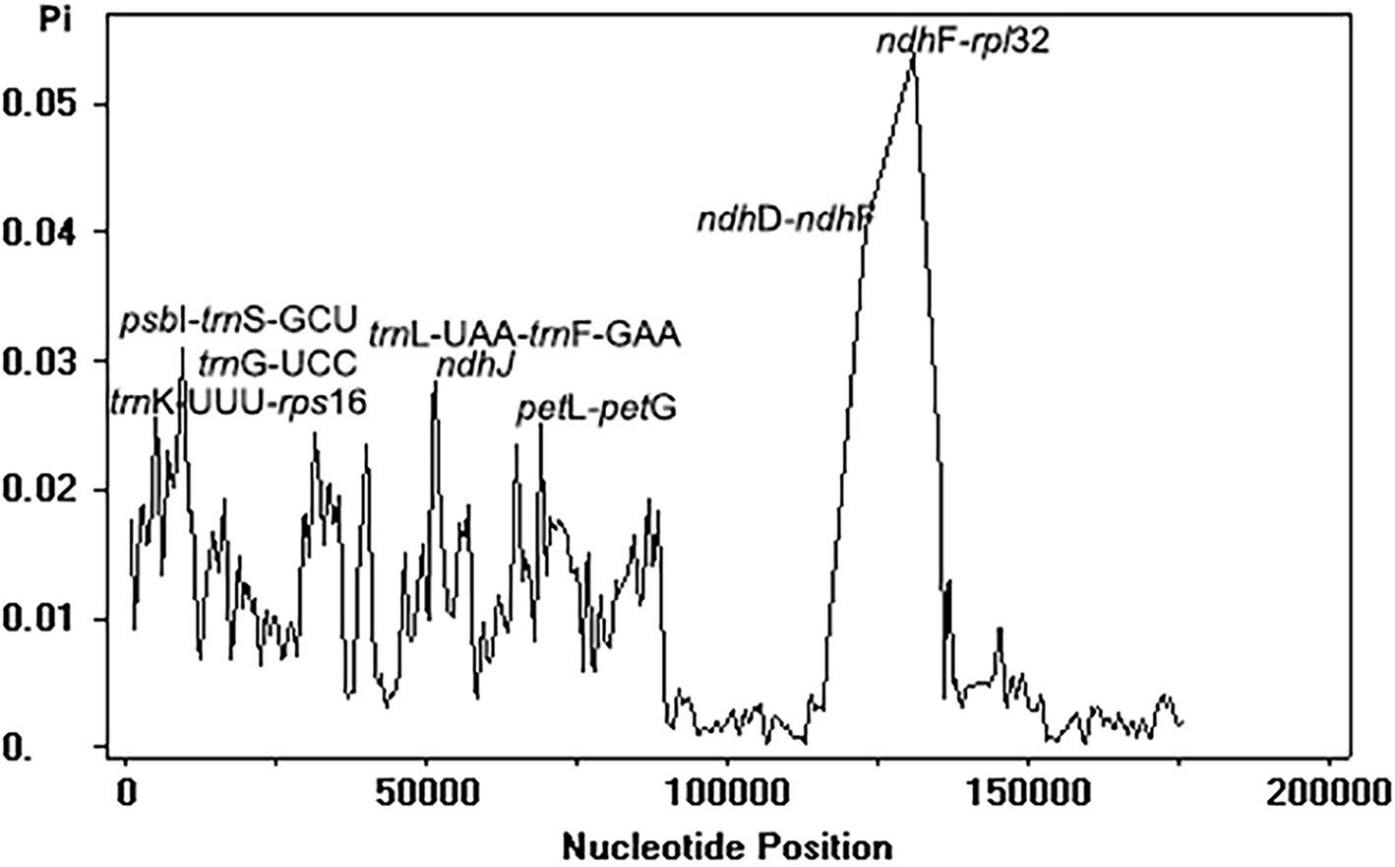
Sliding window analysis of complete plastome sequences among eight species of *Wikstroemia* (window length: 1000 bp; step size: 500 bp).

The plastome sequence alignment of the eight species of *Wikstroemia*, using the *W. chamaedaphne* plastome as a reference, indicated high sequence conservatism across the plastomes of eight species but not in the plastome of *W. indica* (Fig. 4). Overall, the size and gene order of the plastomes in *Wikstroemia* were well conserved, but a distinct large gap was observed beginning within the *ycf*1 gene sequence of the IRa to 5′ region of the *trn*L-UAG in the IRb of *W. indica*. Both single-copy regions were recorded as having greater sequence divergence than the IR region (Fig. 5). With a Pi-value cut-off point of 0.025, eight highly variable gene regions were identified: *ndh*D-*ndh*F, *ndh*F-*rpl*32, *ndh*J, *pet*L-*pet*G, *psb*I-*trn*S-GCU, *trn*G-UCC, *trn*K-UUU-*rps*16 and the *trn*L-UAA-*trn*F-GAA intergenic spacer regions. Six of the highly variable regions were located in the LSC, while two of them were in the SSC region.

### Contraction and expansion in the IR region

**Figure 6:**
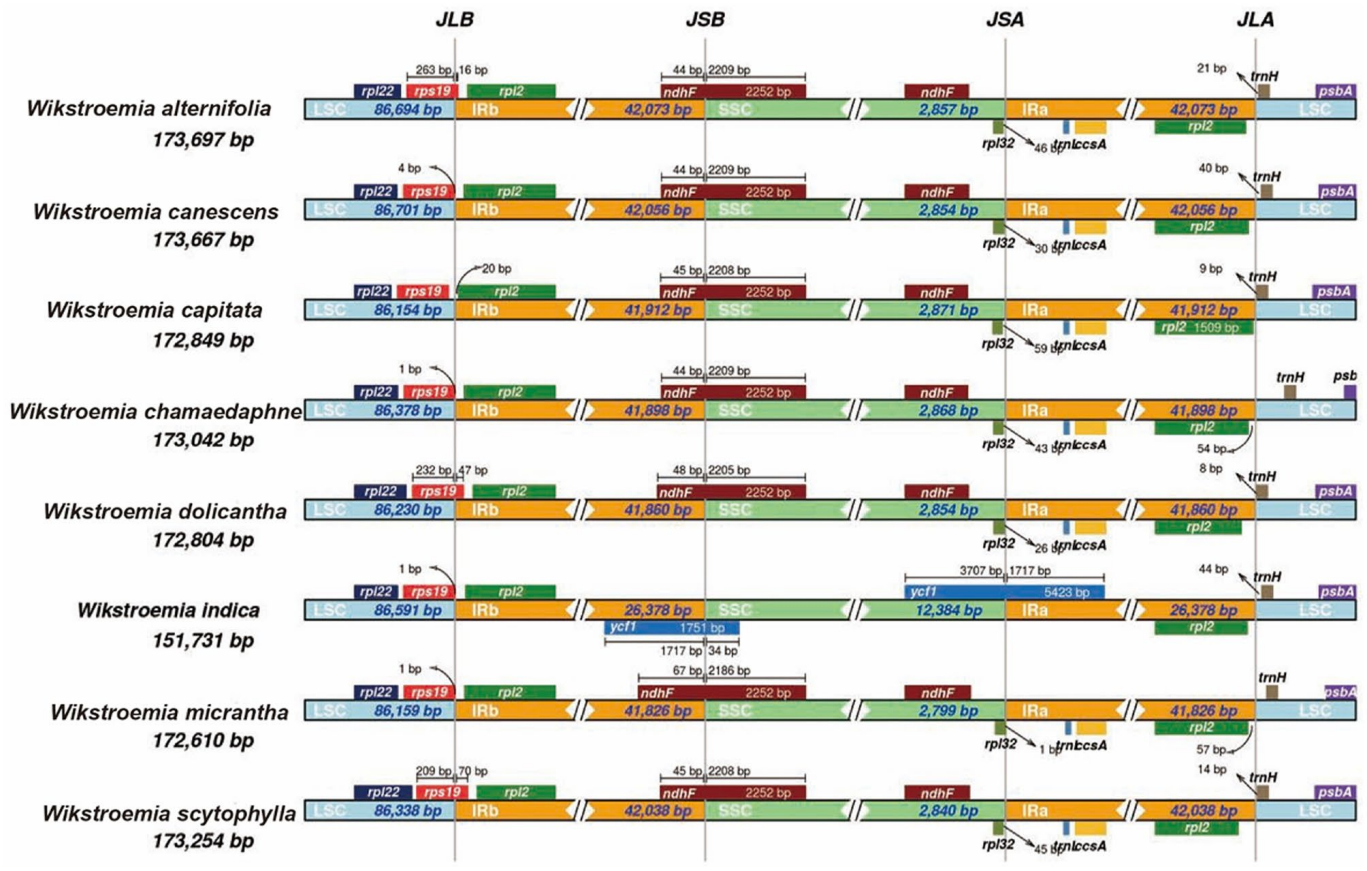
Comparison of borders between LSC, SSC and IR regions across the plastomes of eight species of *Wikstroemia*. Image was generated with IRscope^50^.

Genes adjacent to the IR borders were consistent across members of *Wikstroemia*, except in *W. indica*, which varied in its adjacent genes at the IRb/SSC (JSB) and IRa/SSC (JSA) borders (Fig. 6). In contrast to the *rpl*32 and *ndh*F genes in the SSC region, adjacent to JSB and JSA, respectively, the *ycf*1 gene was located across both JSA and JSB in the plastomes of *W. indica*. The *trn*L-UAG gene was also adjacent to JSA in the SSC region of the *W. indica* plastome. In comparison, six species (*W. alternifolia*, *W. chamaedaphne*, *W. dolicantha*, *W. indica*, *W. micrantha* and *W. scytophylla*) had their *rps*19 gene crossing the IRb/LSC (JLB) border.

### Selection pressure

Sixty-nine shared protein-coding genes were included in the selection pressure analysis between *Aquilaria sinensis* and *W. capitata* (Table 3). When analysed separately, the K_a_/K_s_ values indicated that five genes, namely, *rpl*2, *rps*7, *rps*18, *ycf*1 and *ycf*2, displayed positive selection; 61 genes indicated purifying selection, and three genes did not exhibit any synonymous (K_s_) values indicative of selection due to the constraints of the model used. The K_a_/K_s_ value for the combined dataset revealed that the overall selection pressure of the 69 shared protein-coding genes was 0.435, showing signals of purifying selection.

**Table 3:** Selection pressure analysis of 69 shared protein-coding gene sequences for *Aquilaria sinensis* (GenBank accession MN720647) and *Wikstroemia capitata*, analysed separately and combined.

No.	Gene	K_a_	K_s_	K_a_/K_s_	P-value	Length of alignment (bp)	No. of substitutions
1	*acc*D	0.1378	0.2406	0.5725	0.0039	1254	176
2	*atp*A	0.0201	0.1416	0.1417	0.0000	1518	67
3	*atp*B	0.0073	0.0956	0.0760	0.0000	1494	42
4	*atp*E	0.0203	0.0873	0.2321	0.0083	399	14
5	*atp*H	0.0113	0.1009	0.1116	0.0048	243	8
6	*atp*I	0.0198	0.0651	0.3035	0.0060	741	22
7	*ccs*A	0.0123	0.0269	0.4564	0.1179	969	15
8	*mat*K	0.0580	0.1229	0.4717	0.0003	1512	106
9	*ndh*A	0.2932	0.3161	0.9273	0.7027	2001	488
10	*ndh*B	0.0050	0.0135	0.3699	0.0630	2052	14
11	*ndh*C	0.0137	0.1561	0.0877	0.0000	360	13
12	*ndh*D	0.0091	0.0134	0.6809	0.4815	1518	15
13	*ndh*E	0.0000	0.0355	0.0000	0.0000	297	2
14	*ndh*F	0.0699	0.3206	0.2180	0.0000	2124	216
15	*ndh*G	0.0049	0.0168	0.2923	0.2213	528	4
16	*ndh*H	0.0089	0.0290	0.3052	0.0175	1179	15
17	*ndh*I	0.0073	0.0117	0.6236	0.5313	501	4
18	*ndh*J	0.0027	0.1144	0.0238	0.0000	474	12
19	*ndh*K	0.0271	0.1467	0.1849	0.0000	681	34
20	*pet*A	0.0154	0.0622	0.2475	0.0007	960	25
21	*pet*B	0.0343	0.0618	0.5561	0.0379	1380	55
22	*pet*D	0.0455	0.0623	0.7303	0.2785	1167	56
23	*pet*G	0.0000	0.1517	0.0000	0.0000	108	4
24	*pet*L	0.0000	0.1007	0.0000	0.0000	93	2
25	*pet*N	0.0000	0.0947	0.0000	0.0000	87	2
26	*psa*A	0.0049	0.0877	0.0555	0.0000	2250	57
27	*psa*B	0.0048	0.0849	0.0565	0.0000	2202	50
28	*psa*C	0.0000	0.0199	0.0000	0.0000	243	1
29	*psa*I	0.0242	0.0786	0.3074	0.2424	111	4
30	*psa*J	0.0000	0.0927	0.0000	0.0000	132	3
31	*psb*A	0.0000	0.1473	0.0000	0.0000	1059	36
32	*psb*B	0.0093	0.1145	0.0815	0.0000	1524	46
33	*psb*C	0.0019	0.0659	0.0294	0.0000	1419	26
34	*psb*D	0.0062	0.0539	0.1150	0.0000	1059	18
35	*psb*E	0.0000	0.1038	0.0000	0.0000	249	5
36	*psb*F	0.0122	0.1299	0.0938	0.0234	117	5
37	*psb*H	0.0532	0.1261	0.4222	0.1156	219	14
38	*psb*I	0.0000	0.1344	0.0000	0.0000	108	4
39	*psb*J	0.0221	0.0364	0.6092	0.5452	120	3
40	*psb*K	0.0307	0.0000	NA	0.0565	183	4
41	*psb*L	0.0000	0.0388	0.0000	0.0000	114	1
42	*psb*M	0.0396	0.0433	0.9136	0.6602	102	4
43	*psb*T	0.0000	0.2167	0.0000	0.0000	99	5
44	*psb*Z	0.0000	0.1432	0.0000	0.0000	186	5
45	*rbc*L	0.0100	0.0927	0.1077	0.0000	1431	38
45	*rpl*2	0.0092	0.0048	1.909	0.5022	1407	11
46	*rpl*14	0.0328	0.1322	0.2482	0.0043	366	19
48	*rpl*20	0.0496	0.0838	0.5914	0.3819	348	19
49	*rpl*22	0.0592	0.1671	0.3540	0.0072	501	37
50	*rpl*23	0.0091	0.0000	NA	0.0000	279	2
51	*rpl*32	0.1266	0.4313	0.2936	0.0075	153	22
52	*rpl*33	0.0503	0.1237	0.4068	0.1006	198	13
53	*rpl*36	0.0357	0.0414	0.8627	0.6408	111	4
54	*rpo*A	0.0463	0.1371	0.3378	0.0001	984	61
55	*rpo*B	0.0170	0.1010	0.1686	0.0000	3210	117
56	*rpo*C2	0.0346	0.0965	0.3589	0.0000	4092	190
57	*rps*2	0.0308	0.0555	0.5542	0.1389	708	25
58	*rps*3	0.0493	0.1459	0.3378	0.0011	654	42
59	*rps*4	0.0441	0.0946	0.4656	0.0475	603	32
60	*rps*7	0.0362	0.0320	1.131	0.9772	465	16
61	*rps*8	0.0361	0.0838	0.4301	0.0790	402	18
62	*rps*11	0.0799	0.1749	0.4567	0.0197	414	38
63	*rps*14	0.0351	0.0628	0.5586	0.2622	300	12
64	*rps*15	0.0317	0.0000	NA	0.0073	270	6
65	*rps*18	0.0634	0.0338	1.879	0.5130	276	15
66	*rps*19	0.0496	0.1094	0.4534	0.1955	270	16
67	*ycf*1	0.0746	0.0508	1.469	0.0075	5316	353
68	*ycf*2	0.0424	0.0141	3.007	0.0000	6717	232
69	*ycf*4	0.0232	0.0595	0.3902	0.0567	561	17
70	Concatenated dataset	0.0373	0.0857	0.4350	0.0000	65,172	3019

*NA* not available.

### Phylogenetic analysis

**Figure 7:**
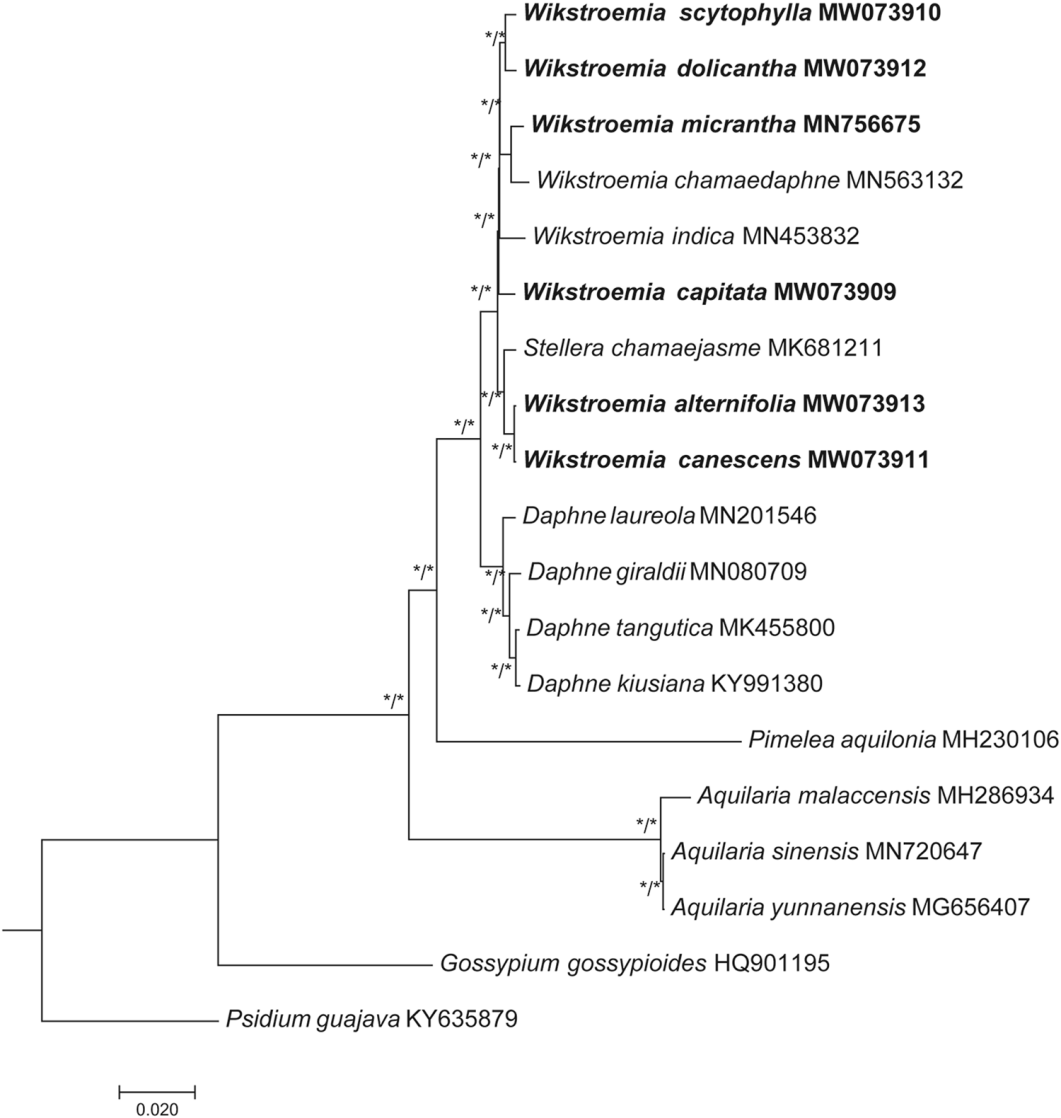
Maximum likelihood (ML) and Bayesian inference (BI) of *Wikstroemia* and allied genera based on the complete plastome sequences excluding the inverted repeat A (IRa) region, and a dataset of the intergenic spacer (IGS) regions of 17 taxa representing 5 genera of Thymelaeaceae, analysed separately. Branch nodes that were calculated with reliable support values (ML: bootstrap ≥ 75%; BI: posterior probability ≥ 0.90) are indicated with an asterisk (*). Sequences obtained through this study are indicated in bold; two species, *Psidium guajava* (KY635879) and *Gossypium gossypioides* (HQ901195), were included as outgroups.

The maximum-likelihood (ML) and Bayesian inference (BI) trees based on the complete plastome sequences excluding the IRa sequences and the dataset of the intergenic spacer (IGS) sequences revealed that all the branch nodes for eight species of *Wikstroemia* included in the phylogenetic tree were supported with high bootstrap values and Bayesian posterior probabilities (ML: ≥ 90%; BI: ≥ 95%) (Fig. 7). For the dataset of the total gene sequences containing protein-coding genes, tRNAs, and rRNAs that are shared by all species, strong posterior probabilities were recorded in most of the branch nodes of the BI tree but not in the ML tree, in which moderate bootstrap support was recorded for the backbone structure of the *Wikstroemia* clade (see Supplementary Figure S1 online). The molecular placement of *W. capitata* and *W. indica*, forming sister to each other under low branch support, in the ML tree and BI tree based on the dataset of all protein-coding genes was incongruent with the phylogenetic trees based on the datasets using complete plastome sequences excluding the IRa and intergenic spacer (IGS) sequences. The ML trees and BI trees based on the datasets of the first, second, and third codons of each amino acid in the protein-coding sequences did not display matching molecular placement of *Wikstroemia* when compared with each other; most of the branches were poorly supported in the *Wikstroemia* clade (see Supplementary Figure S2 online). The phylogenetic tree using complete plastome sequences excluding the IRa sequences suggested that a paraphyletic relationship was present in *Wikstroemia*. Two species, *W. alternifolia* and *W. canescens*, were clustered with *Stellera chamaejasme*, while six species of *Wikstroemia* (*W. capitata*, *W. chamaedaphne*, *W. dolicantha*, *W. indica*, *W. micrantha* and *W. scytophylla*) formed a monophyletic group.

**Figure 8:**
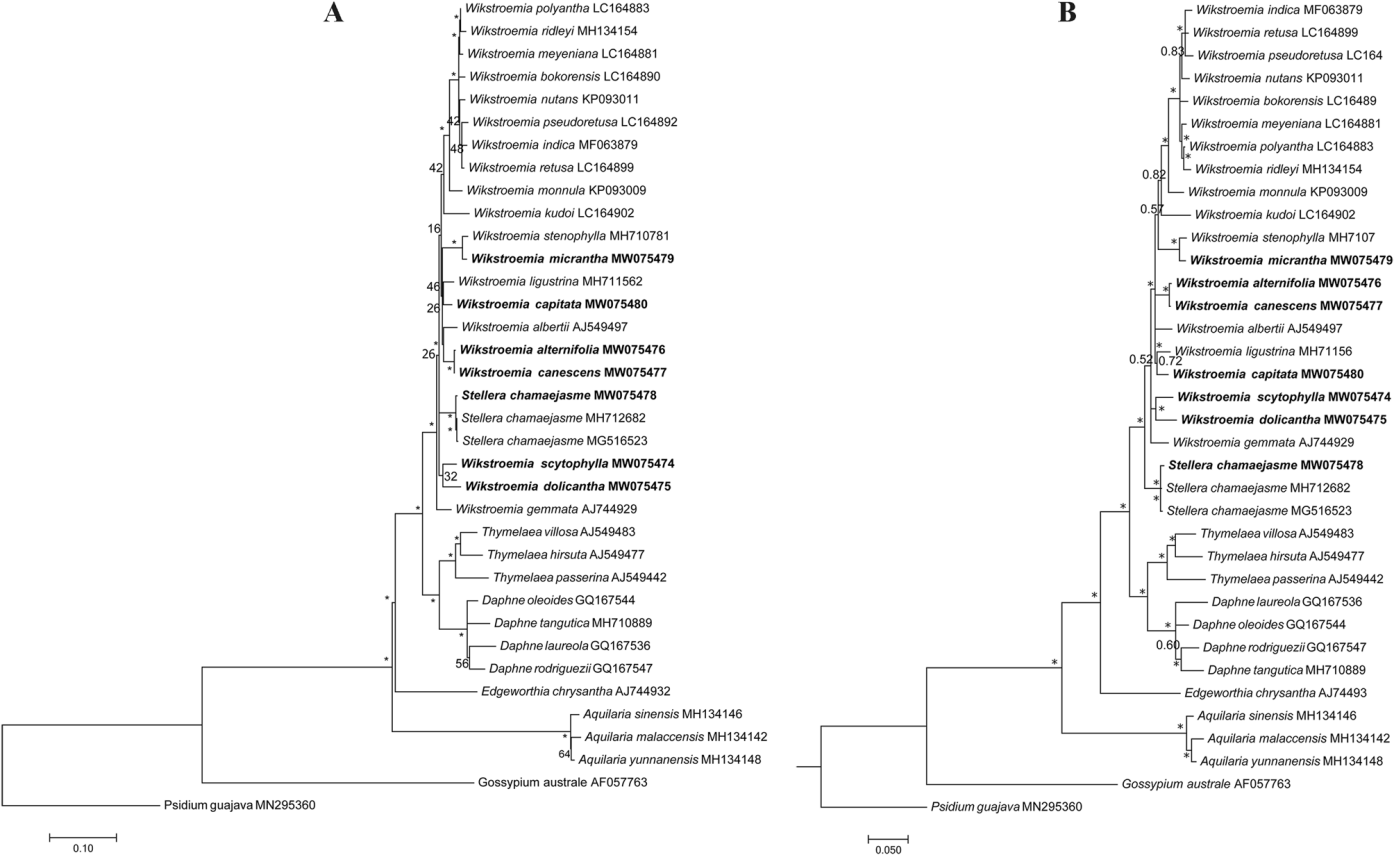
Phylogenetic analyses of Thymelaeaceae based on nuclear ribosomal DNA internal transcribed spacer (ITS) gene sequences of 34 taxa representing 6 genera of Thymelaeaceae. (**A**) Maximum-likelihood (ML) and (**B**) Bayesian inference (BI) tree analyses were conducted with 1000 bootstrap replicates. Branch nodes that were calculated with reliable support values (ML: bootstrap ≥ 75%; BI: posterior probability ≥ 0.90) are indicated with an asterisk (*). Two species, *Psidium guajava* (MN2953604) and *Gossypium australe* (AF057763), were included as outgroups.

The ITS-based ML tree revealed a paraphyletic relationship between *Wikstroemia* and *S. chamaejasme*, while most of the branch nodes within the *Wikstroemia* clade were not highly supported (Fig. 8A). Strong bootstrap support was recorded for the sistership between *W. alternifolia* and *W. canescens* and between *W. micrantha* and *W. stenophylla*. Weakly supported sisterships were present between *W. dolicantha* and *W. scytophylla* and between *W. capitata* and *W. ligustrina*. In contrast, the BI analysis displayed a monophyletic relationship within the *Wikstroemia* clade (Fig. 8B). Similar to the ML tree, sisterships were strongly supported between *W. alternifolia* and *W. canescens* and between *W. micrantha* and *W. stenophylla* but not between *W. dolicantha* and *W. scytophylla* or between *W. capitata* and *W. ligustrina* in the BI tree.

## Discussion

The plastomes of the species in *Wikstroemia* examined in this study were highly conserved, which is similar to the situation in other angiosperms. The length of the plastomes of the six species of *Wikstroemia* varied little and were similar in size to typical angiosperms^25^. The same number and contents of the genes were predicted in this study, suggesting that the evolution of the gene sequences was consistent across the six species. Similar to most angiosperms, sequence repeats for A/T were more abundant than those of G/C in the *Wikstroemia* plastomes and may represent bias in the base composition, which is potentially affected by the tendency of the genome to change to A-T rather than to G-C^26^. An additional validation step for these SSRs, for which five novel SSR primer sets were designed, was conducted for the six species of *Wikstroemia* reported in this study. Details of the newly designed SSR primer sets and the resulting pherograms are included for reference (see Supplementary Table S3 and Data S1 online).

Expansion and contraction of the IR region are major evolutionary events that influence the length of the plastomes^27^. The IR junctions in the plastomes reported in this study were placed and annotated with Geneious Prime^28^ and further validated with GeSeq^29^ as well as Sanger sequencing using novel specific primer sets (see Supplementary Table S4 and Data S2 online). Our study indicated that the contractions and expansions of the IR regions exhibited relatively stable patterns within *Wikstroemia*, with slight variation; gene recombination between the repetitive sequence or poly-A structure and tRNA could be one of the reasons for the change in length in the IR region^30^. However, *W. indica* indicated dissimilarity in its IR borders, which differed from most angiosperms^31^. We suspect that the plastome IR contraction and expansion in *W. indica* is severe and may be due to extensive gene transfer and larger IR expansion due to the results of the double strand break repair mechanism^32–34^. Interestingly, when compared to other species of *Wikstroemia* sequenced in this study, the plastome of *W. indica* was smaller (151,731 bp) and had a greater GC content (37.4%)^8^. We found that the plastome of *W. indica* had a shorter IR region and larger SSC region than other species of *Wikstroemia*. Changes in the placement of the IR borders in the plastome of *W. indica* were likely due to contraction of the IR region, causing a loss in the number and content of the genes. Among the genes that were not found in *W. indica* but were present in other species of *Wikstroemia*, *ndh*A, *ndh*G, and *ndh*I were supposed to be present in the IR region; genes such as *ccs*A, *ndh*D, *ndh*E, *ndh*H, *psa*C, *rps*15, and *trn*L-UAG that are commonly duplicated in the IR regions were reduced to only one copy and were transferred to the SSC region, while the *ndh*F and *rpl*32 genes, common genes in the SSC region, were not detected. Therefore, it can be concluded that the contraction of the IR region that caused gene loss contributed to the difference in plastome content between *W. indica* and the other seven species of *Wikstroemia*.

Molecular evidence based on plastome sequences revealed a nonmonophyletic relationship between the species of *Wikstroemia* due to *W. alternifolia* and *W. canescens* clustering with *Stellera chamaejasme*. Information on the phylogenetic relationships of *Wikstroemia* species is scarce. Although taxonomic work is challenging in a genus with diverse species, continuous efforts among taxonomists studying members of the Thymelaeaceae have provided some insights into the taxonomic status of *Wikstroemia*. To provide better insight into the phylogenetic relationships at the nuclear level, we used ITS sequences to perform ML and BI analyses. Unlike phylogenomic tree analyses on complete plastome sequences, low bootstrap support and Bayesian posterior probabilities were observed at the species level in *Wikstroemia*. The molecular placement of the species of *Wikstroemia*, however, was identical in both the ML and BI trees, while the most distinct difference between both phylogenetic trees was the placement of *S. chamaejasme*. In the ML tree based on the ITS sequences, *S. chamaejasme* clustered within the *Wikstroemia* clade, but it was sister to *Wikstroemia* in the BI tree. The discordance between the plastid and nuclear phylogenies in this study may be due to phylogenetic sorting, convergence, unequal rates of evolution, long branch attraction, and introgression^35^. However, low branch node support in both the ITS-based ML and BI trees suggested that either the inclusion of additional nuclear gene sequences or the application of the restriction site-associated DNA sequence (RAD-Seq) technique that integrates up to 10% of the nuclear genome^36^ could be helpful in resolving the phylogenetic relationships within *Wikstroemia*. Evidently, in this study, the use of a single nuclear gene sequence, i.e., ITS, which was suspected to be useful in delimiting many plants at the species level^37^, was insufficient for resolving the phylogenetic relationships between *Stellera* and *Wikstroemia*.

Members of *Wikstroemia* currently comprise species previously placed under *Capura* L., *Daphne* L., *Diplomorpha* Meisn., *Daphnimorpha* Nakai, *Lonicera* L., *Passerina* L., *Restella* Pobed., and *Stellera* L.^1,38^. The monotypic genus *Stellera*, which exhibits strikingly similar morphological characteristics, has troubled some taxonomists who compared it to *Wikstroemia*. At least five species were placed under *Stellera* before they were transferred to *Wikstroemia*; others were transferred to allied genera, such as *Daphne*, *Diarthron* and *Thymelaea* in the tribe Daphneae^38^. This is understandable, as *Stellera* has a longer taxonomic history, i.e., back to 1747, when compared to other genera in the Daphneae. As a result, *S. chamaejasme*, as the type species, is the only species left in the genus. Based on the literature, we found that *Wikstroemia* has an interesting nomenclatural history in which two genera, *Diplomorpha* and *Daphnimorpha*, were synonymized and excluded. Combining *Stellera* with *Wikstroemia* was previously proposed by transferring the type species *S. chamaejasme* to the monotypic subgenus *Chamaejasme*^11,39^. However, the proposal was rejected, as *Stellera* has priority over *Wikstroemia*^40^, and based on the Rules of Nomenclature, the combination can only be accepted if *Stellera* is proposed as a nomen genus rejiciendum (nom. gen. rejic.)^12^. Therefore, we do not exclude the possibility that *Stellera* should be synonymized with *Wikstroemia*. In that case, *Wikstroemia* would be synonymized under *Stellera*. One should not jump to such a conclusion rashly, based on the current situation, as the taxonomic dispute on whether *Wikstroemia* should be synonymized with *Daphne* is yet unresolved^41^. Unless *Daphne* is considered in a subsequent taxonomic treatment, based on the phylogenetic trees in this study, we could only conclude that *Wikstroemia* is not monophyletic and that *Stellera* is unquestionably closely related to *Wikstroemia*.

While phylogenetic analyses based on the plastome sequences of *Wikstroemia* have proven to be promising, we suggest that larger sampling is required to resolve the taxonomic dispute in *Wikstroemia* through a molecular approach. We foresee that the genetic information in the complete plastome sequences of *Wikstroemia* is deemed sufficient and could aid in the classification of *Wikstroemia*, both at the genus level and at the species level.

## Conclusion

To the best of our knowledge, this study presents the first genome-scale analysis of species of *Wikstroemia*. The findings revealed high conservation of genes in the plastomes. The identification of highly variable gene regions in the plastome sequences of *Wikstroemia* could potentially be useful in resolving phylogenetic relationships in the genus. A strong sistership between *Wikstroemia* and the monotypic genus *Stellera* was present. The ML and BI trees based on the plastome sequences revealed that all the branch nodes for eight species of *Wikstroemia* included in the phylogenetic tree were supported with high bootstrap values and Bayesian posterior probabilities (ML: ≥ 90%; BI: ≥ 95%), while the ITS-based tree analyses could not properly resolve the phylogenetic relationship between *Stellera* and *Wikstroemia*. Nevertheless, the molecular data obtained in this study will serve as a valuable resource for providing greater insights into the taxonomy and phylogeny of Thymelaeaceae.

## Materials and methods

### Plant materials and DNA extraction

Fresh leaf materials of six species of *Wikstroemia*, *W. alternifolia*, *W. canescens*, *W. capitata*, *W. dolicantha*, *W. micrantha* and *W. scytophylla,* were collected from botanical gardens and natural populations in China (Table 1). Species identification was carried out by Yonghong Zhang, and the voucher specimens were deposited in the Herbarium of Yunnan Normal University (YNUB)^42^. Based on the local guidelines and legislation on plant study, permissions for collections and research were unnecessary, as the samples were not collected in protected areas or recorded as threatened species. However, *W. scytophylla* was collected under permit record number w2021005, which was authorized in the Kunming Botanical Garden, Chinese Academy of Science, China. All collections are permitted and legal. Total genomic DNA was extracted using the Axygen AxyPrep Multisource Genomic Miniprep DNA kit (Corning, USA) following the manufacturer’s protocol.

### Plastome sequencing, assembly and annotation

A sequence library was constructed, and sequencing was performed on the Illumina HiSeq 2500-PE150 platform (Illumina, USA). All raw reads were filtered using NGS QC Toolkit version 2.3.3 with default parameters to obtain clean reads^43^. The plastome was de novo assembled using NOVOPlasty^44^ with the *rbc*L gene sequence of *Daphne kiusiana* (GenBank accession KY991380) as the seed sequence. Gene annotation was performed in Geneious Prime^28^ using the complete plastome sequence of *W. chamaedaphne* (GenBank accession MN563132) as the reference genome. The circular physical map of the plastome was generated using OGDRAW^45^.

### Repeat analyses

SSRs were identified using MISA-web^46^, in which parameters for the identification of perfect mono-, di-, tri-, tetra-, penta-, and hexanucleotide motifs were set for a minimum of 10, 5, 4, 3, 3, and 3 repeats, respectively. Long repeats, including forward, palindrome, reverse and complement repeats, were determined using REPuter^47^ with a Hamming distance of 3 and a minimal repeat size of 30 bp.

### Codon usage

Coding sequences of each plastome were extracted, and the RSCU was analysed using MEGA7^48^.

### Comparative genome and divergence analyses

The complete plastome sequences of two species of *Wikstroemia*, *W. chamaedaphne* (GenBank accession MN563132) and *W. indica* (GenBank accession MN453832), which were available in NCBI GenBank, were downloaded and included in subsequent analyses. By using the plastome sequences of *W. chamaedaphne* as the reference genome, nucleotide variation in the plastome sequence alignment of the eight species of *Wikstroemia* was visualized using mVISTA^49^ in Shuffle-LAGAN mode. To detect the expansion and contraction of the IR region in the plastomes across the eight species, the IR/SC boundaries of the plastomes were visualized using IRscope^50^. To detect the mutational hotspots and divergence regions in the plastomes of the eight species, sequence alignment of the plastome sequences was carried out using Geneious Prime^28^. Calculations of the nucleotide variability (Pi) among the eight plastomes were performed using DnaSP v5^51^ with a window length of 1000 bp and a step size of 500 bp.

### Selection pressure analysis

The ratio of nonsynonymous to synonymous substitutions (K_a_/K_s_) of protein-coding genes was calculated for *Aquilaria sinensis* (GenBank accession MN720647) and *Wikstroemia capitata*. Calculations were conducted for two sets of data: (1) shared genes analysed separately and (2) a combined dataset containing all shared genes. Prior to sequence alignment using MUSCLE embedded in MEGA7^48^, the plastome sequence of *A. sinensis* was reannotated to ensure uniformity. For the combined dataset, the coding sequences were concatenated manually. Selection pressure acting on these genes was estimated using KaKs_Calculator 2.0^52^ based on the Yang and Nielsen codon frequency (YN) model, with parameters for the initial ratio of transition to transversion frequency (K) set between 0.3 and 0.7. A K_a_/K_s_ value equal to or less than 1.0 indicates the presence of purifying selection, in which changes in gene residues of amino acids that may favour excess synonymous versus nonsynonymous substitutions have been avoided, while the presence of positive selection is specified if the K_a_/K_s_ value is more than 1.0.

### Polymerase chain reaction and Sanger sequencing

Polymerase chain reaction (PCR) amplification was carried out in a 20 µL reaction volume using the ITS universal primer set: 5F: 5ʹ-GGAAGTAAAAGTCGTAA-CAAGG-3ʹ (forward) and 4R: 5ʹ-TCCTCCGCTTATTGATATGC-3ʹ (reverse). The PCRs for the nuclear ribosomal DNA ITS region contained 10 µL of 2× Taq PCR Starmix with loading dye (Genstar Biosolutions, China), 0.4 µM of each primer and 20 ng of genomic DNA as a template. PCR amplifications were conducted on a T100 Thermal Cycler (Bio-Rad, USA), with initial denaturation at 93 °C for 5 min; 40 cycles of denaturation at 93 °C for 30 s, annealing at 60 °C for 30 s, and extension at 72 °C for 30 s; and a final extension at 72 °C for 5 min. PCR products were sent for direct Sanger sequencing at both ends using an ABI 3730 DNA Analyzer (Applied Biosystems, USA).

### Phylogenetic analyses

Phylogenetic analyses were conducted based on the plastome or gene sequences of 17 selected taxa from Thymelaeaceae. Two species, *Psidium guajava* (Myrtaceae; GenBank accession KY635879) and *Gossypium gossypioides* (Malvaceae; GenBank accession HQ901195), were included as outgroups. Seven datasets, including the (1) complete plastome sequences excluding IRa, (2) the total gene sequences containing protein-coding genes, tRNAs, and rRNAs that are shared by all species, (3) the intergenic spacer (IGS) sequences, (4) all protein-coding genes that are shared by all species, and (5) three additional subdatasets at the codon level for the first/second/third codons of each amino acid in the protein-coding sequences, were used to perform phylogenetic inferences. Part of the complete plastome sequences excluding the Ira and the targeted genic and intergenic regions in the plastomes, was extracted and concatenated using Geneious Prime^28^, while the first/second/third codons of each amino acid in the shared genes were extracted using MEGA7^48^. Sequence alignment was carried out using MAFFT v7.450^53^. The ML tree was constructed based on all the sequence datasets using RAxML 8.2.11^54^. The general-time-reversible (GTR) and gamma distributed (+ G) (+ GTR + G) DNA substitution model was selected, and all branch nodes were calculated under 1000 bootstrap replicates. BI analysis was conducted for all the datasets^54,55^. BI analysis was executed through the MrBayes^55^ pipeline available in the CIPRESS Science Gateway web portal^56^. Markov chain Monte Carlo (MCMC) was conducted with 2,000,000 generations, and sampling was collected every 100 cycles. The final tree was visualized using FigTree^57^ and edited manually.

The ITS sequences were aligned and manually trimmed for their primer sequences to obtain clean sequences. A total of 26 additional ITS sequences derived from members of the Thymelaeaceae were downloaded from the NCBI GenBank and MUSCLE-aligned against the ITS sequences of the six species of *Wikstroemia* used in this study using MEGA7^48^. Two species, *P. guajava* (Myrtaceae; GenBank accession MN295360) and *Gossypium australe* (Malvaceae; GenBank accession AF057763), were included as outgroups. The alignment was trimmed using trimAL v1.2^58^ with the gappyout method to reduce systematic errors produced by poor alignment. The optimal DNA substitution model for the ML analysis using the “Find Best DNA/Protein Model (ML)” function embedded in MEGA7^48^ was calculated to be the Kimura two-parameter (K2P) model with the discrete Gamma model (+ G4) and invariant sites included (+ I) (= K2P + G + I). ML analysis was performed using MEGA7^48^ with 1000 bootstrap replicates. BI analysis was conducted with a previously described method^55^.

## Supplementary Information

Supplementary Information 1.

Supplementary Information 2.

## Data Availability

The complete chloroplast sequences generated and analysed in this paper are available in GenBank (https://www.ncbi.nlm.nih.gov/genbank/, accession numbers listed in the text).
