# Supplementary material for: Complete plastomes of six species of Wikstroemia (Thymelaeaceae) reveal paraphyly with the monotypic genus Stellera
Source: Sci Rep. 2021 Jun 30;11:13608. doi: 10.1038/s41598-021-93057-3 (PMC8245458; doi:10.1038/s41598-021-93057-3)
Supplement: Supplementary file 1 — Supplementary Information 1. [file 41598_2021_93057_MOESM1_ESM.docx]

**Supplementary Materials**

Figure S1 Phylogenetic inference of *Wikstroemia* and allied genera based on the (A) dataset of the total gene sequences containing protein-coding genes, tRNAs, and rRNAs, and (B) dataset of all protein-coding genes that are shared by all species, using maximum-likelihood (ML) and Bayesian inference (BI) methods, was analysed separately. Branch nodes that were calculated with reliable support values (ML: bootstrap ≥75%; BI: posterior probability ≥0.90) are indicated with an asterisk (*).

Figure S2 Maximum-likelihood (ML) and Bayesian inference (BI) analyses of the datasets of the (A) first, (B) second, and (C) third codons of each amino acid in the protein-coding sequences of *Wikstroemia* and allied genera, analysed separately. Branch nodes that were calculated with reliable support values (ML: bootstrap ≥75%; BI: posterior probability ≥0.90) are indicated with an asterisk (*).

Data S1 Sanger sequencing files corresponding to each SSR.

Data S2 Sanger sequencing files corresponding to the validation of inverted repeat boundaries.
