# Supplementary material for: Complete plastomes of six species of Wikstroemia (Thymelaeaceae) reveal paraphyly with the monotypic genus Stellera
Source: Sci Rep. 2021 Jun 30;11:13608. doi: 10.1038/s41598-021-93057-3 (PMC8245458; doi:10.1038/s41598-021-93057-3)
Supplement: Supplementary file 2 — Supplementary Information 2. [file 41598_2021_93057_MOESM2_ESM.zip › Table S1.docx]

Table S1: Information of introns and exons for protein-coding genes in six *Wikstroemia* species used in this study.

| Species | Gene | Location | Exon І (bp) | Intron І (bp) | Exon ІІ (bp | Intron ІІ (bp) | Exon ІІІ (bp) |
| --- | --- | --- | --- | --- | --- | --- | --- |
| 1. *alternifolia* | *atp*F | LSC | 411 | 915 | 144 | na | na |
|  | *pet*B | LSC | 6 | 766 | 698 | na | na |
|  | *pet*D | LSC | 8 | 795 | 475 | na | na |
|  | *rpl*16 | LSC | 399 | 1069 | 9 | na | na |
|  | *rpo*C1 | LSC | 1617 | 796 | 432 | na | na |
|  | *rps*16 | LSC | 40 | 927 | 215 | na | na |
|  | *trn*G-UCC | LSC | 23 | 721 | 49 | na | na |
|  | *trn*K-UUU | LSC | 35 | 2505 | 37 | na | na |
|  | *trn*L-UAA | LSC | 34 | 540 | 50 | na | na |
|  | *trn*V-UAC | LSC | 35 | 599 | 38 | na | na |
|  | *ycf*3 | LSC | 153 | 757 | 228 | 741 | 126 |
|  | *ndh*A | IR | 558 | 1158 | 540 | na | na |
|  | *ndh*B | IR | 756 | 676 | 777 | na | na |
|  | rpl2 | IR | 434 | 694 | 393 | na | na |
|  | *trn*A-UGC | IR | 38 | 819 | 35 | na | na |
|  | *trn*I-GAU | IR | 37 | 960 | 35 | na | na |
| *W. canescens* | *atp*F | LSC | 411 | 923 | 144 | na | na |
|  | *pet*B | LSC | 6 | 766 | 698 | na | na |
|  | *pet*D | LSC | 8 | 799 | 475 | na | na |
|  | *rpl*16 | LSC | 399 | 1069 | 9 | na | na |
|  | *rpo*C1 | LSC | 1617 | 794 | 432 | na | na |
|  | *rps*16 | LSC | 40 | 925 | 215 | na | na |
|  | *trn*G-UCC | LSC | 23 | 726 | 49 | na | na |
|  | *trn*K-UUU | LSC | 35 | 2507 | 37 | na | na |
|  | *trn*L-UAA | LSC | 34 | 540 | 50 | na | na |
|  | *trn*V-UAC | LSC | 35 | 618 | 38 | na | na |
|  | *ycf*3 | LSC | 153 | 757 | 228 | 742 | 126 |
|  | *ndh*A | IR | 558 | 1159 | 540 | na | na |
|  | *ndh*B | IR | 756 | 686 | 777 | na | na |
|  | *rpl*2 | IR | 434 | 694 | 393 | na | na |
|  | *trn*A-UGC | IR | 38 | 818 | 35 | na | na |
|  | *trn*I-GAU | IR | 37 | 960 | 35 | na | na |
| *W. capitata* | *atp*F | LSC | 411 | 911 | 144 | na | na |
|  | *pet*B | LSC | 6 | 754 | 698 | na | na |
|  | *pet*D | LSC | 8 | 784 | 475 | na | na |
|  | *rpl*16 | LSC | 399 | 1110 | 9 | na | na |
|  | *rpo*C1 | LSC | 1617 | 792 | 432 | na | na |
|  | *rps*16 | LSC | 200 | 937 | 40 | na | na |
|  | *trn*G-UCC | LSC | 23 | 718 | 49 | na | na |
|  | *trn*K-UUU | LSC | 35 | 2500 | 37 | na | na |
|  | *trn*L-UAA | LSC | 34 | 534 | 50 | na | na |
|  | *trn*V-UAC | LSC | 35 | 597 | 38 | na | na |
|  | *ycf*3 | LSC | 153 | 771 | 228 | 742 | 126 |
|  | *ndh*A | IR | 558 | 1155 | 540 | na | na |
|  | *ndh*B | IR | 756 | 687 | 777 | na | na |
|  | *rpl*2 | IR | 434 | 684 | 393 | na | na |
|  | *trn*A-UGC | IR | 38 | 818 | 35 | na | na |
|  | *trn*I-GAU | IR | 37 | 960 | 35 | na | na |
| *W. dolicantha* | *atp*F | LSC | 411 | 912 | 144 | na | na |
|  | *pet*B | LSC | 6 | 755 | 698 | na | na |
|  | *pet*D | LSC | 8 | 783 | 475 | na | na |
|  | *rpl*16 | LSC | 399 | 1070 | 9 | na | na |
|  | *rpo*C1 | LSC | 1617 | 784 | 432 | na | na |
|  | *rps*16 | LSC | 242 | 922 | 40 | na | na |
|  | *trn*G-UCC | LSC | 23 | 717 | 49 | na | na |
|  | *trn*K-UUU | LSC | 35 | 2498 | 37 | na | na |
|  | *trn*L-UAA | LSC | 34 | 539 | 50 | na | na |
|  | *trn*V-UAC | LSC | 35 | 599 | 38 | na | na |
|  | *ycf*3 | LSC | 153 | 765 | 228 | 740 | 126 |
|  | *ndh*A | IR | 558 | 1154 | 540 | na | na |
|  | *ndh*B | IR | 756 | 686 | 777 | na | na |
|  | *rp*l2 | IR | 434 | 682 | 393 | na | na |
|  | *trn*A-UGC | IR | 38 | 819 | 35 | na | na |
|  | *trn*I-GAU | IR | 37 | 965 | 35 | na | na |
| 1. *micrantha* | *atp*F | LSC | 411 | 898 | 144 | na | na |
|  | *pet*B | LSC | 6 | 765 | 698 | na | na |
|  | *pet*D | LSC | 8 | 784 | 475 | na | na |
|  | *rpl*16 | LSC | 399 | 1085 | 9 | na | na |
|  | *rpo*C1 | LSC | 1617 | 790 | 432 | na | na |
|  | *rps*16 | LSC | 212 | 926 | 40 | na | na |
|  | *trn*G-UCC | LSC | 23 | 717 | 49 | na | na |
|  | *trn*K-UUU | LSC | 38 | 2508 | 37 | na | na |
|  | *trn*L-UAA | LSC | 34 | 536 | 50 | na | na |
|  | *trn*V-UAC | LSC | 35 | 597 | 38 | na | na |
|  | *ycf*3 | LSC | 153 | 759 | 228 | 738 | 126 |
|  | *ndh*A | IR | 558 | 1147 | 540 | na | na |
|  | *ndh*B | IR | 756 | 687 | 777 | na | na |
|  | *rpl*2 | IR | 434 | 682 | 393 | na | na |
|  | *trn*A-UGC | IR | 38 | 820 | 35 | na | na |
|  | *trn*I-GAU | IR | 37 | 960 | 35 | na | na |
| *W. scytophylla* | *atp*F | LSC | 411 | 893 | 144 | na | na |
|  | *pet*B | LSC | 6 | 756 | 698 | na | na |
|  | *pet*D | LSC | 8 | 782 | 475 | na | na |
|  | *rpl*16 | LSC | 399 | 1076 | 9 | na | na |
|  | *rpo*C1 | LSC | 1617 | 783 | 432 | na | na |
|  | *rps*16 | LSC | 215 | 933 | 40 | na | na |
|  | *trn*G-UCC | LSC | 23 | 725 | 49 | na | na |
|  | *trn*K-UUU | LSC | 35 | 2500 | 37 | na | na |
|  | *trn*L-UAA | LSC | 34 | 550 | 50 | na | na |
|  | *trn*V-UAC | LSC | 35 | 592 | 38 | na | na |
|  | *ycf*3 | LSC | 153 | 768 | 228 | 740 | 126 |
|  | *ndh*A | IR | 558 | 1153 | 540 | na | na |
|  | *ndh*B | IR | 756 | 687 | 777 | na | na |
|  | *rpl*2 | IR | 434 | 686 | 393 | na | na |
|  | *trn*A-UGC | IR | 38 | 819 | 35 | na | na |
|  | *trn*I-GAU | IR | 37 | 960 | 35 | na | na |

Note: na=not available
