# Supplementary material for: Complete plastomes of six species of Wikstroemia (Thymelaeaceae) reveal paraphyly with the monotypic genus Stellera
Source: Sci Rep. 2021 Jun 30;11:13608. doi: 10.1038/s41598-021-93057-3 (PMC8245458; doi:10.1038/s41598-021-93057-3)
Supplement: Supplementary file 2 — Supplementary Information 2. [file 41598_2021_93057_MOESM2_ESM.zip › Table S2.docx]

Table S2: Relative synonymous codon usage of protein-coding genes in chloroplast genomes of six *Wikstroemia* species used in this study. RSCU that are recorded more than (>) 1.00 were indicated in bold.

| Amino acid | Codon | *W. alternifolia* | | *W. canescens* | | *W. capitata* | | *W. dolicantha* | | *W. micrantha* | | *W. scytophylla* | |
| --- | --- | --- | --- | --- | --- | --- | --- | --- | --- | --- | --- | --- | --- |
|  |  | Count | RSCU | Count | RSCU | Count | RSCU | Count | RSCU | Count | RSCU | Count | RSCU |
| Ala | GCU | 629 | **1.70** | 660 | **1.71** | 637 | **1.72** | 639 | **1.73** | 630 | **1.71** | 541 | **1.72** |
|  | GCC | 257 | 0.69 | 268 | 0.69 | 261 | 0.7 | 254 | 0.69 | 263 | 0.71 | 223 | 0.71 |
|  | GCA | 433 | **1.17** | 451 | **1.17** | 413 | **1.11** | 419 | **1.14** | 413 | **1.12** | 354 | **1.13** |
|  | GCG | 164 | 0.44 | 169 | 0.44 | 172 | 0.46 | 164 | 0.44 | 169 | 0.46 | 139 | 0.44 |
| Arg | CGU | 365 | **1.25** | 377 | **1.25** | 356 | **1.23** | 362 | **1.24** | 365 | **1.26** | 313 | **1.19** |
|  | CGC | 123 | 0.42 | 128 | 0.42 | 123 | 0.42 | 124 | 0.43 | 118 | 0.41 | 121 | 0.46 |
|  | CGA | 397 | **1.36** | 406 | **1.35** | 392 | **1.35** | 387 | **1.33** | 394 | **1.36** | 343 | **1.30** |
|  | CGG | 124 | 0.42 | 131 | 0.43 | 130 | 0.45 | 132 | 0.45 | 126 | 0.44 | 123 | 0.47 |
|  | AGA | 545 | **1.86** | 559 | **1.85** | 542 | **1.87** | 545 | **1.87** | 534 | **1.85** | 494 | **1.87** |
|  | AGG | 201 | 0.69 | 210 | 0.7 | 200 | 0.69 | 198 | 0.68 | 198 | 0.68 | 190 | 0.72 |
| Asn | AAU | 1049 | **1.54** | 1118 | **1.55** | 1036 | **1.54** | 1046 | **1.55** | 1047 | **1.54** | 956 | **1.54** |
|  | AAC | 311 | 0.46 | 325 | 0.45 | 309 | 0.46 | 308 | 0.45 | 310 | 0.46 | 287 | 0.46 |
| Asp | GAU | 886 | **1.56** | 953 | **1.56** | 873 | **1.55** | 879 | **1.56** | 879 | **1.56** | 803 | **1.56** |
|  | GAC | 252 | 0.44 | 267 | 0.44 | 251 | 0.45 | 247 | 0.44 | 247 | 0.44 | 229 | 0.44 |
| Cys | UGU | 237 | **1.44** | 252 | **1.43** | 236 | **1.44** | 236 | **1.44** | 235 | **1.43** | 191 | **1.40** |
|  | UGC | 92 | 0.56 | 100 | 0.57 | 92 | 0.56 | 91 | 0.56 | 93 | 0.57 | 82 | 0.6 |
| Gln | CAA | 785 | **1.54** | 823 | **1.54** | 782 | **1.54** | 785 | **1.55** | 788 | **1.55** | 718 | **1.54** |
|  | CAG | 235 | 0.46 | 249 | 0.46 | 231 | 0.46 | 228 | 0.45 | 230 | 0.45 | 217 | 0.46 |
| Glu | GAA | 1122 | **1.47** | 1177 | **1.47** | 1115 | **1.48** | 1120 | **1.48** | 1127 | **1.48** | 999 | **1.46** |
|  | GAG | 402 | 0.53 | 420 | 0.53 | 390 | 0.52 | 394 | 0.52 | 394 | 0.52 | 366 | 0.54 |
| Gly | GGU | 606 | **1.29** | 626 | **1.27** | 601 | **1.28** | 604 | **1.28** | 603 | **1.28** | 526 | **1.31** |
|  | GGC | 188 | 0.4 | 204 | 0.41 | 182 | 0.39 | 186 | 0.4 | 184 | 0.39 | 157 | 0.39 |
|  | GGA | 730 | **1.55** | 773 | **1.57** | 740 | **1.58** | 740 | **1.57** | 739 | **1.57** | 604 | **1.51** |
|  | GGG | 359 | 0.76 | 371 | 0.75 | 353 | 0.75 | 353 | 0.75 | 352 | 0.75 | 317 | 0.79 |
| His | CAU | 519 | **1.53** | 542 | **1.53** | 518 | **1.55** | 518 | **1.53** | 519 | **1.53** | 474 | **1.54** |
|  | CAC | 161 | 0.47 | 166 | 0.47 | 152 | 0.45 | 159 | 0.47 | 159 | 0.47 | 143 | 0.46 |
| Ile | AUU | 1191 | **1.46** | 1270 | **1.48** | 1170 | **1.46** | 1183 | **1.46** | 1184 | **1.46** | 988 | **1.48** |
|  | AUC | 474 | 0.58 | 491 | 0.57 | 475 | 0.59 | 480 | 0.59 | 479 | 0.59 | 401 | 0.60 |
|  | AUA | 776 | 0.95 | 818 | 0.95 | 766 | 0.95 | 761 | 0.94 | 769 | 0.95 | 619 | 0.92 |
| Leu | UUA | 964 | **1.90** | 1039 | **1.93** | 961 | **1.91** | 972 | **1.92** | 970 | **1.92** | 774 | **1.84** |
|  | UUG | 611 | **1.20** | 642 | **1.19** | 605 | **1.20** | 610 | **1.20** | 609 | **1.20** | 535 | **1.27** |
|  | CUU | 659 | **1.30** | 702 | **1.30** | 649 | **1.29** | 643 | **1.27** | 642 | **1.27** | 534 | **1.27** |
|  | CUC | 205 | 0.40 | 212 | 0.39 | 196 | 0.39 | 200 | 0.39 | 200 | 0.4 | 173 | 0.41 |
|  | CUA | 415 | 0.82 | 432 | 0.80 | 409 | 0.81 | 418 | 0.82 | 416 | 0.82 | 343 | 0.82 |
|  | CUG | 194 | 0.38 | 202 | 0.38 | 197 | 0.39 | 197 | 0.39 | 198 | 0.39 | 159 | 0.38 |
| Lys | AAA | 1210 | **1.48** | 1269 | **1.48** | 1199 | **1.48** | 1212 | **1.48** | 1208 | **1.48** | 1118 | **1.46** |
|  | AAG | 428 | 0.52 | 444 | 0.52 | 420 | 0.52 | 422 | 0.52 | 428 | 0.52 | 411 | 0.54 |
| Met | AUG | 661 | 1.00 | 702 | 1.00 | 648 | 1.00 | 656 | 1.00 | 657 | 1.00 | 527 | 1.00 |
| Phe | UUU | 1133 | **1.31** | 1226 | **1.33** | 1110 | **1.31** | 1127 | **1.31** | 1123 | **1.31** | 947 | **1.30** |
|  | UUC | 592 | 0.69 | 617 | 0.67 | 582 | 0.69 | 590 | 0.69 | 594 | 0.69 | 510 | 0.70 |
| Pro | CCU | 433 | **1.48** | 460 | **1.50** | 439 | **1.51** | 433 | **1.49** | 436 | **1.49** | 377 | **1.48** |
|  | CCC | 234 | 0.80 | 242 | 0.79 | 225 | 0.78 | 232 | 0.80 | 235 | 0.80 | 214 | 0.84 |
|  | CCA | 332 | **1.14** | 346 | **1.13** | 334 | **1.15** | 334 | **1.15** | 330 | **1.13** | 272 | **1.07** |
|  | CCG | 170 | 0.58 | 178 | 0.58 | 163 | 0.56 | 166 | 0.57 | 169 | 0.58 | 154 | 0.61 |
| Ser | UCU | 620 | **1.73** | 684 | **1.77** | 610 | **1.72** | 621 | **1.73** | 611 | **1.71** | 566 | **1.79** |
|  | UCC | 338 | 0.95 | 351 | 0.91 | 335 | 0.94 | 335 | 0.93 | 343 | 0.96 | 302 | 0.95 |
|  | UCA | 424 | **1.19** | 457 | **1.18** | 425 | **1.20** | 432 | **1.21** | 429 | **1.20** | 361 | **1.14** |
|  | UCG | 217 | 0.61 | 228 | 0.59 | 216 | 0.61 | 213 | 0.59 | 215 | 0.60 | 176 | 0.56 |
|  | AGU | 402 | **1.12** | 451 | 1.16 | 398 | **1.12** | 406 | **1.13** | 407 | **1.14** | 363 | **1.15** |
|  | AGC | 145 | 0.41 | 153 | 0.40 | 146 | 0.41 | 143 | 0.40 | 145 | 0.40 | 134 | 0.42 |
| Thr | ACU | 558 | **1.62** | 598 | **1.63** | 553 | **1.63** | 556 | **1.63** | 556 | **1.62** | 498 | **1.68** |
|  | ACC | 252 | 0.73 | 257 | 0.70 | 251 | 0.74 | 249 | 0.73 | 251 | 0.73 | 218 | 0.74 |
|  | ACA | 426 | **1.24** | 460 | **1.26** | 416 | **1.23** | 423 | **1.24** | 422 | **1.23** | 356 | **1.20** |
|  | ACG | 140 | 0.41 | 150 | 0.41 | 138 | 0.41 | 138 | 0.4 | 142 | 0.41 | 112 | 0.38 |
| Trp | UGG | 512 | 1.00 | 534 | 1.00 | 502 | 1.00 | 508 | 1.00 | 511 | 1.00 | 427 | 1.00 |
| Tyr | UAU | 854 | **1.61** | 911 | **1.61** | 847 | **1.61** | 860 | **1.62** | 846 | **1.60** | 716 | **1.61** |
|  | UAC | 208 | 0.39 | 219 | 0.39 | 206 | 0.39 | 203 | 0.38 | 213 | 0.4 | 175 | 0.39 |
| Val | GUU | 554 | **1.48** | 580 | **1.49** | 543 | **1.46** | 556 | **1.48** | 553 | **1.48** | 448 | **1.41** |
|  | GUC | 181 | 0.48 | 183 | 0.47 | 188 | 0.51 | 183 | 0.49 | 183 | 0.49 | 153 | 0.48 |
|  | GUA | 577 | **1.54** | 603 | **1.55** | 569 | **1.53** | 574 | **1.53** | 570 | **1.52** | 493 | **1.55** |
|  | GUG | 187 | 0.50 | 191 | 0.49 | 187 | 0.5 | 192 | 0.51 | 193 | 0.52 | 180 | 0.57 |
| End | UAA | 43 | **1.45** | 45 | **1.45** | 42 | **1.43** | 41 | **1.38** | 43 | **1.45** | 39 | **1.50** |
|  | UAG | 25 | 0.84 | 27 | 0.87 | 25 | 0.85 | 26 | 0.88 | 25 | 0.84 | 21 | 0.81 |
|  | UGA | 21 | 0.71 | 21 | 0.68 | 21 | 0.72 | 22 | 0.74 | 21 | 0.71 | 18 | 0.69 |
