# Supplementary material for: Complete plastomes of six species of Wikstroemia (Thymelaeaceae) reveal paraphyly with the monotypic genus Stellera
Source: Sci Rep. 2021 Jun 30;11:13608. doi: 10.1038/s41598-021-93057-3 (PMC8245458; doi:10.1038/s41598-021-93057-3)
Supplement: Supplementary file 2 — Supplementary Information 2. [file 41598_2021_93057_MOESM2_ESM.zip › Table S3.docx]

Table S3 Details of the novel microsatellite markers and results of the short sequence repeats obtained from six species of the *Wikstroemia*

| Locus | Primer sequence (5’–3’) | Primer location on the cp genome(bp) | Repeat motif | T_a_* (◦C) | Species | Corresponding Sanger sequencing files for each SSR |
| --- | --- | --- | --- | --- | --- | --- |
| SSR1 | F: TGTTCAACAGCTCCCCTTCG  R: TTTTGTCAGAAGAATCCTCCGA | 13,172  13,602 | (AT)^10^ | 60.3  57.4 | *W. alternifolia* | 17-RXK30_SSR1_F_TSS20210203-0871-0279_A04.ab1 |
|  |  |  |  |  | *W. canescens* | 17-RXK32_SSR1_F_TSS20210203-0871-0279_B04.ab1 |
|  |  |  |  |  | *W. capitata* | 17-RXK33_SSR1_F_TSS20210203-0871-0279_C04.ab1 |
|  |  |  |  |  | *W. dolicantha* | 17-RXK39_SSR1_F_TSS20210203-0871-0279_D04.ab1 |
|  |  |  |  |  | *W. micrantha* | 17-RXK43_SSR1_F_TSS20210203-0871-0279_E04.ab1 |
|  |  |  |  |  | *W. scytophylla* | 17-RXK48_SSR1_F_TSS20210203-0871-0279_F04.ab1 |
| SSR5 | F: GGGTTCGATTCCCGCTATCC  R: CAGACATGTTTGTTGCCGGG | 39,888  40,370 | (TAAT)^3^ | 60.3  60.0 | *W. alternifolia* | 23-RXK30_SSR5_F_TSS20210203-0871-0279_E08.ab1 |
|  |  |  |  |  | *W. canescens* | 23-RXK32_SSR5_F_TSS20210203-0871-0279_F08.ab1 |
|  |  |  |  |  | *W. capitata* | 23-RXK33_SSR5_F_TSS20210203-0871-0279_G08.ab1 |
|  |  |  |  |  | *W. dolicantha* | 23-RXK39_SSR5_F_TSS20210203-0871-0279_H08.ab1 |
|  |  |  |  |  | *W. micrantha* | 23-RXK43_SSR5_F_TSS20210203-0871-0279_A09.ab1 |
|  |  |  |  |  | *W. scytophylla* | 23-RXK48_SSR5_F_TSS20210203-0871-0279_B09.ab1 |
| SSR6 | F: GTGGTAGAGTAACGCCATGGT  R: GACAGATTCTTGCGGGGTCA | 34,384  34,966 | (T)^10^ | 59.8  60.0 | *W. alternifolia* | 24-RXK30_SSR6_F_TSS20210203-0871-0279_C09.ab1 |
|  |  |  |  |  | *W. canescens* | 24-RXK32_SSR6_F_TSS20210203-0871-0279_D09.ab1 |
|  |  |  |  |  | *W. capitata* | 24-RXK33_SSR6_F_TSS20210203-0871-0279_E09.ab1 |
|  |  |  |  |  | *W. dolicantha* | 24-RXK39_SSR6_F_TSS20210203-0871-0279_F09.ab1 |
|  |  |  |  |  | *W. micrantha* | 24-RXK43_SSR6_F_TSS20210203-0871-0279_G09.ab1 |
|  |  |  |  |  | *W. scytophylla* | 24-RXK48_SSR6_F_TSS20210203-0871-0279_H09.ab1 |
| SSR9 | F: CTTCCCTTCTTGGATCGGGG  R: AGGGTAAGCTGTTGAGAGTTGT | 63,608  64,074 | (TAAT)^3^ | 59.8  59.3 | *W. alternifolia* | 27-RXK30_SSR9_F_TSS20210203-0871-0279_A01.ab1 |
|  |  |  |  |  | *W. canescens* | 27-RXK32_SSR9_F_TSS20210203-0871-0279_B01.ab1 |
|  |  |  |  |  | *W. capitata* | 27-RXK33_SSR9_F_TSS20210203-0871-0279_C01.ab1 |
|  |  |  |  |  | *W. dolicantha* | 27-RXK39_SSR9_F_TSS20210203-0871-0279_D01.ab1 |
|  |  |  |  |  | *W. micrantha* | 27-RXK43_SSR9_F_TSS20210203-0871-0279_E01.ab1 |
|  |  |  |  |  | *W. scytophylla* | 27-RXK48_SSR9_F_TSS20210203-0871-0279_F01.ab1 |
| SSR10 | F: CCGCCTTACCTCGACAGTTA  R: TGCCCAGTAACTCACGTGTG | 24,803  25,287 | (T)^10^ | 59.2  60.3 | *W. alternifolia* | 28-RXK30_SSR10_F_TSS20210203-0871-0279_A02.ab1 |
|  |  |  |  |  | *W. canescens* | 28-RXK32_SSR10_F_TSS20210203-0871-0279_B02.ab1 |
|  |  |  |  |  | *W. capitata* | 28-RXK33_SSR10_F_TSS20210203-0871-0279_C02.ab1 |
|  |  |  |  |  | *W. dolicantha* | 28-RXK39_SSR10_F_TSS20210203-0871-0279_D02.ab1 |
|  |  |  |  |  | *W. micrantha* | 28-RXK43_SSR10_F_TSS20210203-0871-0279_E02.ab1 |
|  |  |  |  |  | *W. scytophylla* | 28-RXK48_SSR10_F_TSS20210203-0871-0279_F02.ab1 |

*Note: Ta = annealing temperature
