# Supplementary material for: Complete plastomes of six species of Wikstroemia (Thymelaeaceae) reveal paraphyly with the monotypic genus Stellera
Source: Sci Rep. 2021 Jun 30;11:13608. doi: 10.1038/s41598-021-93057-3 (PMC8245458; doi:10.1038/s41598-021-93057-3)
Supplement: Supplementary file 2 — Supplementary Information 2. [file 41598_2021_93057_MOESM2_ESM.zip › Table S4.docx]

Table S4 Details of specific primer sets and validation files for inverted repeat (IR) boundaries of six species of *Wikstroemia*

| Locus | Boundary | Primer name | Primer sequence (5’–3’) | Ta (◦C)* | Species name | Corresponding Sanger sequencing files for each SSR |
| --- | --- | --- | --- | --- | --- | --- |
| Junction1 | LSC-IRb | Jone_Cf  Jone_Cr | ATGGCCGATCATTGTGGGTA  AAGTGGGGAATGTTGGGGTG | 67.2  67.9 | *W. alternifolia* | 3-RXK30_JONE-CF_TSS20210203-0871-0279_A03.ab1 |
|  |  |  |  |  | *W. canescens* | 3-RXK32_JONE-CF_TSS20210203-0871-0279_B03.ab1 |
|  |  |  |  |  | *W. capitata* | 3-RXK33_JONE-CF_TSS20210203-0871-0279_C03.ab1 |
|  |  |  |  |  | *W. dolicantha* | 3-RXK39_JONE-CF_TSS20210203-0871-0279_D03.ab1 |
|  |  |  |  |  | *W. micrantha* | 3-RXK43_JONE-CF_TSS20210203-0871-0279_E03.ab1 |
|  |  |  |  |  | *W. scytophylla* | 3-RXK48_JONE-CF_TSS20210203-0871-0279_F03.ab1 |
| Junction2 | IRb-SSC | Jtwo_Cf  Jtwo_Cr | AAAGGTCTCTTTCCCAATTGGG  TCGATAGGCAAACAATCGATGG | 66.0  67.7 | *W. alternifolia* | 7-RXK30_JTWO-CF_TSS20210203-0871-0279_A06.ab1 |
|  |  |  |  |  | *W. canescens* | 7-RXK32_JTWO-CF_TSS20210203-0871-0279_B06.ab1 |
|  |  |  |  |  | *W. capitata* | 7-RXK33_JTWO-CF_TSS20210203-0871-0279_C06.ab1 |
|  |  |  |  |  | *W. dolicantha* | 7-RXK39_JTWO-CF_TSS20210203-0871-0279_D06.ab1 |
|  |  |  |  |  | *W. micrantha* | 7-RXK43_JTWO-CF_TSS20210203-0871-0279_E06.ab1 |
|  |  |  |  |  | *W. scytophylla* | 7-RXK48_JTWO-CF_TSS20210203-0871-0279_F06.ab1 |
| Junction3 | SSC-IRa | Jthree_Af  Jthree_ABCDr | AGAAAGGGTATTGGGCAGCC  AAAGGTCTCTTTCCCAATTGGGT | 66.1  66.5 | *W. alternifolia* | 9-RXK30_JTHREE-AF_TSS20210203-0871-0279_A07.ab1 |
|  |  |  |  |  | *W. canescens* | 9-RXK32_JTHREE-AF_TSS20210203-0871-0279_B07.ab1 |
|  |  |  |  |  | *W. capitata* | 9-RXK33_JTHREE-AF_TSS20210203-0871-0279_C07.ab1 |
|  |  |  |  |  | *W. dolicantha* | 9-RXK39_JTHREE-AF_TSS20210203-0871-0279_D07.ab1 |
|  |  |  |  |  | *W. micrantha* | 9-RXK43_JTHREE-AF_TSS20210203-0871-0279_E07.ab1 |
|  |  |  |  |  | *W. scytophylla* | 9-RXK48_JTHREE-AF_TSS20210203-0871-0279_F07.ab1 |
| Junction4 | IRa-LSC | Jfour_Af  Jfour_Ar | CCAAAAACTGCTCGGCAACA  AAAGAGGGGCGGATGTAGC | 59.9  59.5 | *W. alternifolia* | 13-RXK30_JFOUR-AF_TSS20210203-0871-0279_A01.ab1 |
|  |  |  |  |  | *W. canescens* | 13-RXK32_JFOUR-AF_TSS20210203-0871-0279_B01.ab1 |
|  |  |  |  |  | *W. capitata* | 13-RXK33_JFOUR-AF_TSS20210203-0871-0279_C01.ab1 |
|  |  |  |  |  | *W. dolicantha* | 13-RXK39_JFOUR-AF_TSS20210203-0871-0279_D01.ab1 |
|  |  |  |  |  | *W. micrantha* | 13-RXK43_JFOUR-AF_TSS20210203-0871-0279_E01.ab1 |
|  |  |  |  |  | *W. scytophylla* | 13-RXK48_JFOUR-AF_TSS20210203-0871-0279_F01.ab1 |

*Note: Ta = annealing temperature
