# Supplementary figures and images for: Complete plastomes of six species of Wikstroemia (Thymelaeaceae) reveal paraphyly with the monotypic genus Stellera
Source: Sci Rep. 2021 Jun 30;11:13608. doi: 10.1038/s41598-021-93057-3 (PMC8245458; doi:10.1038/s41598-021-93057-3)

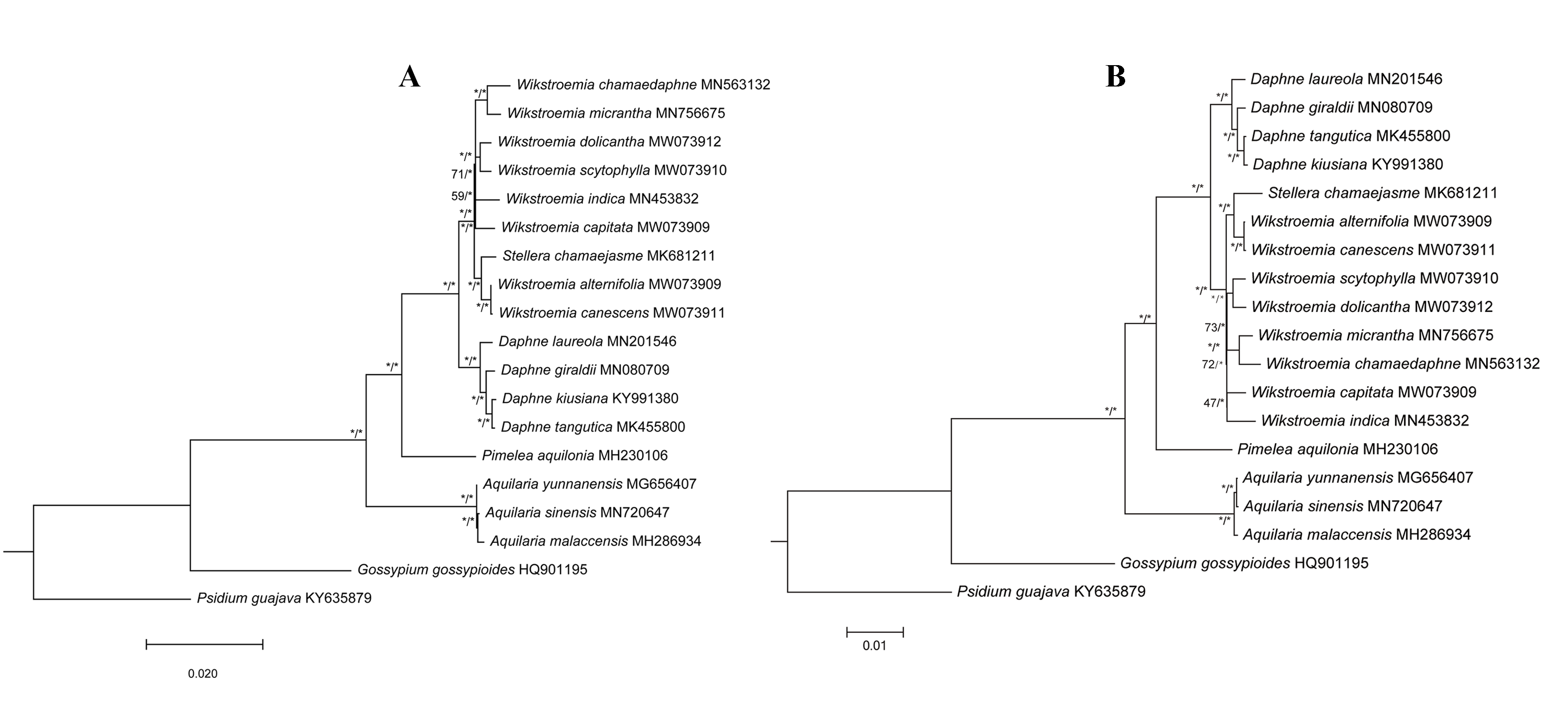

Supplement: Supplementary file 2 — Supplementary Information 2. [file 41598_2021_93057_MOESM2_ESM.zip › Figure S1.tif]

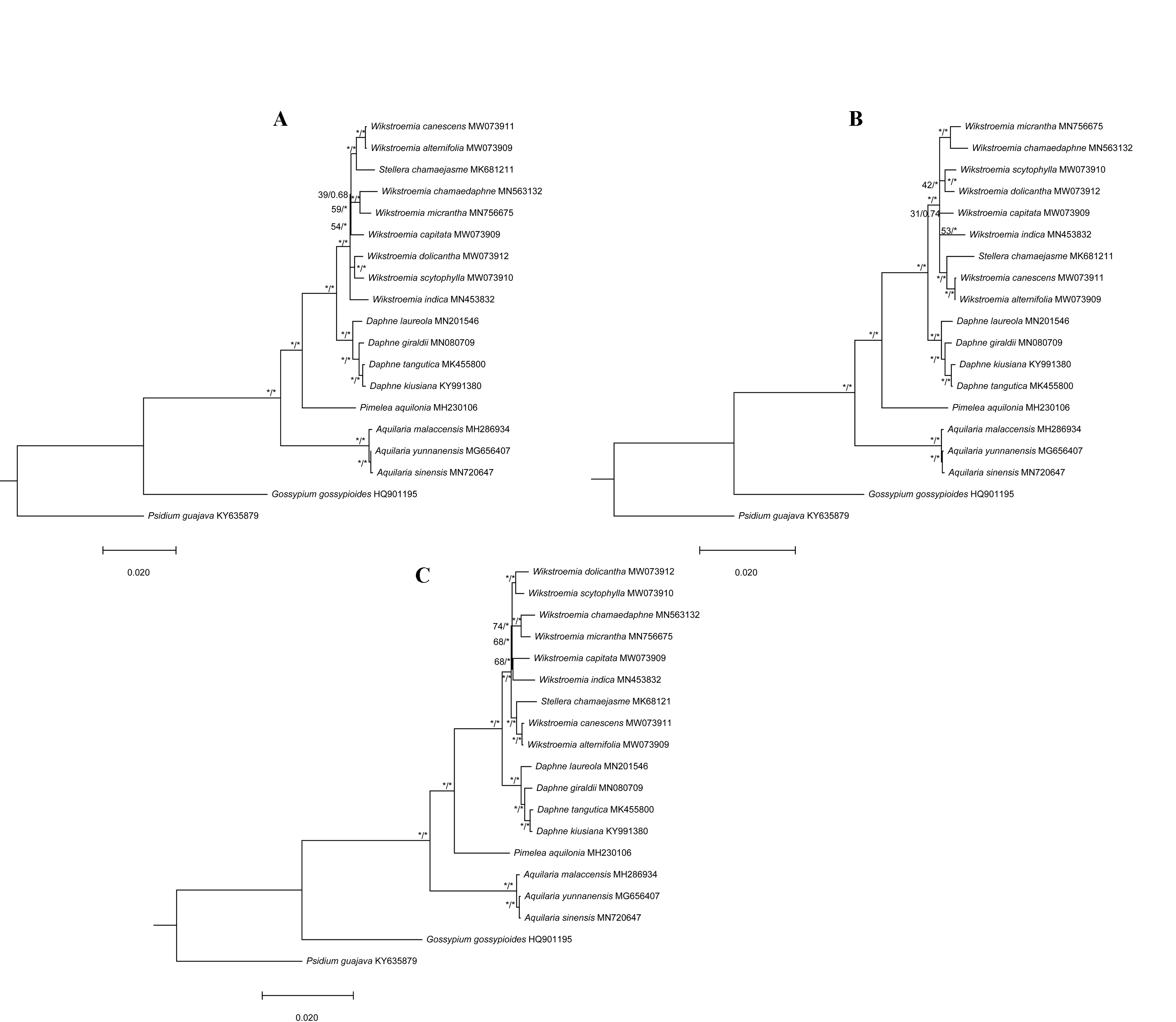

Supplement: Supplementary file 2 — Supplementary Information 2. [file 41598_2021_93057_MOESM2_ESM.zip › Figure S2.tif]
